# Oligopeptide Signaling through *Tb*GPR89 Drives Trypanosome Quorum Sensing

**DOI:** 10.1016/j.cell.2018.10.041

**Published:** 2019-01-10

**Authors:** Federico Rojas, Eleanor Silvester, Julie Young, Rachel Milne, Mabel Tettey, Douglas R. Houston, Malcolm D. Walkinshaw, Irene Pérez-Pi, Manfred Auer, Helen Denton, Terry K. Smith, Joanne Thompson, Keith R. Matthews

**Affiliations:** 1 Institute for Immunology and Infection Research, School of Biological Sciences, University of Edinburgh, Edinburgh EH9 3FL, UK; 2 Institute of Quantitative Biology, Biochemistry and Biotechnology, School of Biological Sciences, University of Edinburgh, Edinburgh EH9 3BF, UK; 3 School of Biology, BSRC, University of St. Andrews, North Haugh, St. Andrews, Fife KY16 9ST, UK

**Keywords:** *Trypanosome brucei*, parasite, quorum sensing, stumpy induction factor, differentiation, GPR89, oligopeptide, sleeping sickness

## Abstract

Trypanosome parasites control their virulence and spread by using quorum sensing (QS) to generate transmissible “stumpy forms” in their host bloodstream. However, the QS signal “stumpy induction factor” (SIF) and its reception mechanism are unknown. Although trypanosomes lack G protein-coupled receptor signaling, we have identified a surface GPR89-family protein that regulates stumpy formation. *Tb*GPR89 is expressed on bloodstream “slender form” trypanosomes, which receive the SIF signal, and when ectopically expressed, *Tb*GPR89 drives stumpy formation in a SIF-pathway-dependent process. Structural modeling of *Tb*GPR89 predicts unexpected similarity to oligopeptide transporters (POT), and when expressed in bacteria, *Tb*GPR89 transports oligopeptides. Conversely, expression of an *E. coli* POT in trypanosomes drives parasite differentiation, and oligopeptides promote stumpy formation *in vitro*. Furthermore, the expression of secreted trypanosome oligopeptidases generates a paracrine signal that accelerates stumpy formation *in vivo*. Peptidase-generated oligopeptide QS signals being received through *Tb*GPR89 provides a mechanism for both trypanosome SIF production and reception.

## Highlights

• Trypanosomes use quorum sensing to differentiate to transmissible stumpy forms
• A GPR89 protein with oligopeptide transport activity drives parasite differentiation
• Oligopeptide mixtures and synthetic di- and tripeptides promote stumpy formation
• Released parasite oligopeptidases generate the paracrine quorum sensing signal *in vivo*

## Graphical Abstract

**Figure:**
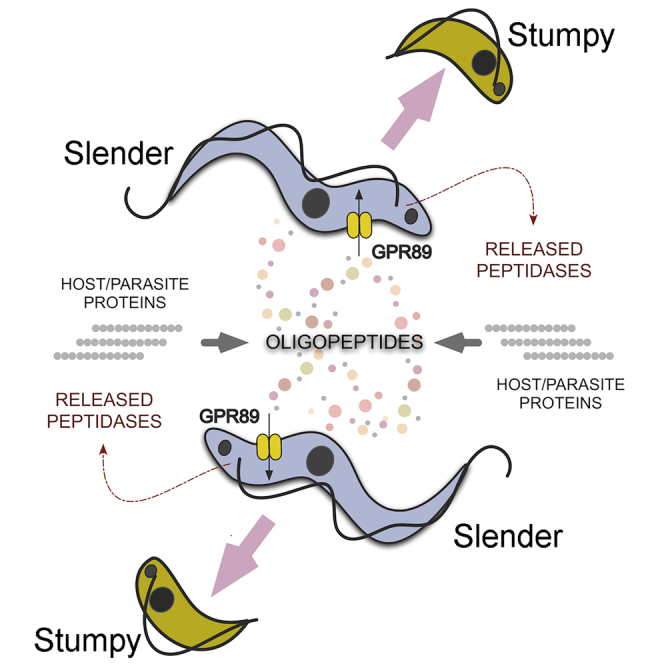

## Teaser

The trypanosome “stumpy” form, important for parasite transmissibility and controlling virulence, is induced by paracrine oligopeptide signaling via an orphan G protein-coupled receptor-related protein functioning as a peptide transporter.

## Introduction

G protein-coupled receptors (GPCRs) and other multipass-transmembrane proteins allow eukaryotic cells to perceive an enormous diversity of extracellular signals, enabling their response to environmental information. Conventionally, GPCRs signal through trimeric G proteins to activate intracellular signaling pathways and are an intense target of drug development for the pharmaceutical industry (Gutierrez and McDonald, 2018). However, GPCRs and their cognate signaling components are not ubiquitous throughout eukaryota, being absent in red and green algae, some chromalveolates, and most excavata (Bradford et al., 2013). Excavates contain a wide variety of important eukaryotic microbial pathogens, including the kinetoplastida comprising *Leishmania* and *Trypanosoma* parasites. Of these, *Trypanosoma brucei spp.*, causing human and animal trypanosomiasis, live extracellularly in the bloodstream of their mammalian host and exploit environmental information to regulate their virulence and transmissibility. Specifically, morphologically “slender form” bloodstream trypanosomes proliferate until signaled to undergo development to non-proliferative “stumpy forms” adapted for transmission (MacGregor et al., 2012). This is a quorum-sensing (QS) type response triggered by the accumulation of a “stumpy induction factor” (SIF), although the nature and mechanism of signaling remains unknown (Reuner et al., 1997, Vassella et al., 1997). Recently, components of the SIF response pathway were uncovered by a genome-wide RNAi screen that identified signal transduction components and gene expression regulators controlling stumpy formation (Mony et al., 2014). However, molecules at the cell surface that detect or transport SIF, or act at early steps in the signaling pathway, remain completely unknown, as is the SIF signal that drives QS.

In plants, GTG1 and GTG2, members of the GPR89 protein family that are classified as orphan GPCRs, detect the extracellular phytohormone abscisic acid (Pandey et al., 2009). In mammalian cells, GPR89 acts as an anion channel protein that is involved in Golgi pH homeostasis (GPHR, Golgi pH regulator) (Maeda et al., 2008). More recent studies suggest GPR89 family members have a location in the Golgi and ER in *Dictyostelium* (Deckstein et al., 2015), with mutants exhibiting developmental defects and perturbed secretory function. In each case, the GPR89 proteins have 9 transmembrane domains (TMDs), distinct from the 7 TMDs conventionally found in GPCRs, and structural bioinformatics analysis has supported the distinction of plant GTGs from the GPCR family (Taddese et al., 2014). Representatives of GPR89 are found in each of the currently recognized supergroups, although they appear to be missing in some organisms, including certain species of fungi and the pathogenic apicomplexan *Cryptosporidium*.

Here, we report the presence of a GPR89 representative, *Tb*GPR89, in the kinetoplastid parasite, *Trypanosoma brucei*. This surface protein is expressed on the parasite stage that receives the QS-signal and can drive stumpy formation via the SIF signaling pathway. African trypanosomes lack conventional oligopeptide transporters, but we show that *Tb*GPR89 can transport oligopeptides, which promote stumpy formation *in vitro*. Furthermore, the expression of secreted oligopeptidases by trypanosomes generates a paracrine signal to co-infecting trypanosomes, driving premature stumpy formation *in vivo*. Our data invoke oligopeptide signals received via *Tb*GPR89 as the long-sought mechanism of trypanosome quorum sensing. These findings provide a novel therapeutic target for trypanosomes that is potentially refractory to the emergence and spread of resistance.

## Results

### *Tb*927.8.1530 Encodes a GPR89 Family Protein

**Figure S1:**
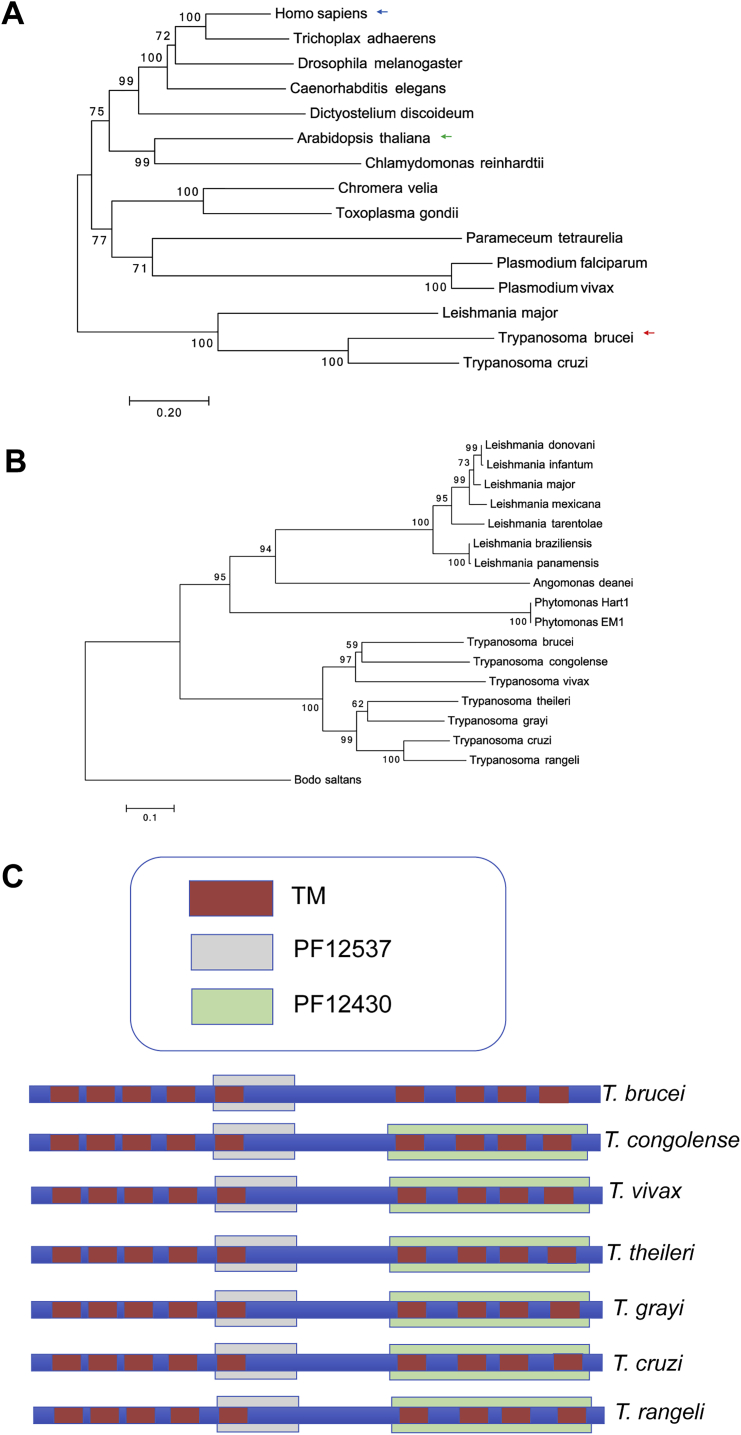
GPR89 Family Members in Kinetoplastid Organisms, Related to Figure 1 (A) Phylogenetic tree of GPR89 family representatives in eukaryota. Human GPR89, *Arabidopsis* GTG1/GTG2 and *Trypanosoma* GPR89 are highlighted. The optimal tree with the sum of branch length = 7.35 is shown. The analysis involved 15 amino acid sequences. All positions containing gaps and missing data were eliminated. There was a total of 383 positions in the final dataset. Accession numbers for each species are; *H. sapiens*, NP_001091081; *T. adhaerens*, XM_002112150; *D. melanogaster*; NP_611016; *C. elegans,*NP_499588; *D.discoideum*, XM_633754; *A thaliana* GTG1, NP_001031235; *C. reinhardtii*, XM_001695842; *C. velia*, Cvel_25352; *T. gondii*, TGME49_286490; *P. tartaurelia,*XM_001426347; *P. falciparum*, PF3D7_1008500; *P. vivax,* PV_094620 *; L. major,* LmjF_07.0330. (B) Phylogenetic tree of GPR89 family representatives in the kinetoplastids. The optimal tree with the sum of branch length = 4.48 is shown. The percentage of replicate trees in which the associated taxa clustered together in the bootstrap test (1000 replicates) are shown next to the branches (Felsenstein, 1981). The tree is drawn to scale, with branch lengths in the same units as those of the evolutionary distances used to infer the phylogenetic tree. The analysis involved 18 amino acid sequences. All positions containing gaps and missing data were eliminated. There are a total of 302 positions in the final dataset. The tree is shown rooted on the *Bodo saltans* GPR89 sequence. *B. saltans* is a free-living nonparasitic marine kinetoplastid of the bodonid clade from which trypanosomatids descended (Jackson et al., 2016). (C) Domain structure of GPR89 members in the kinetoplastida highlighting the position of predicted transmembrane domains (red) Pfam domain 12537 (gray) and Pfam domain 12430 (green).

**Figure 1:**
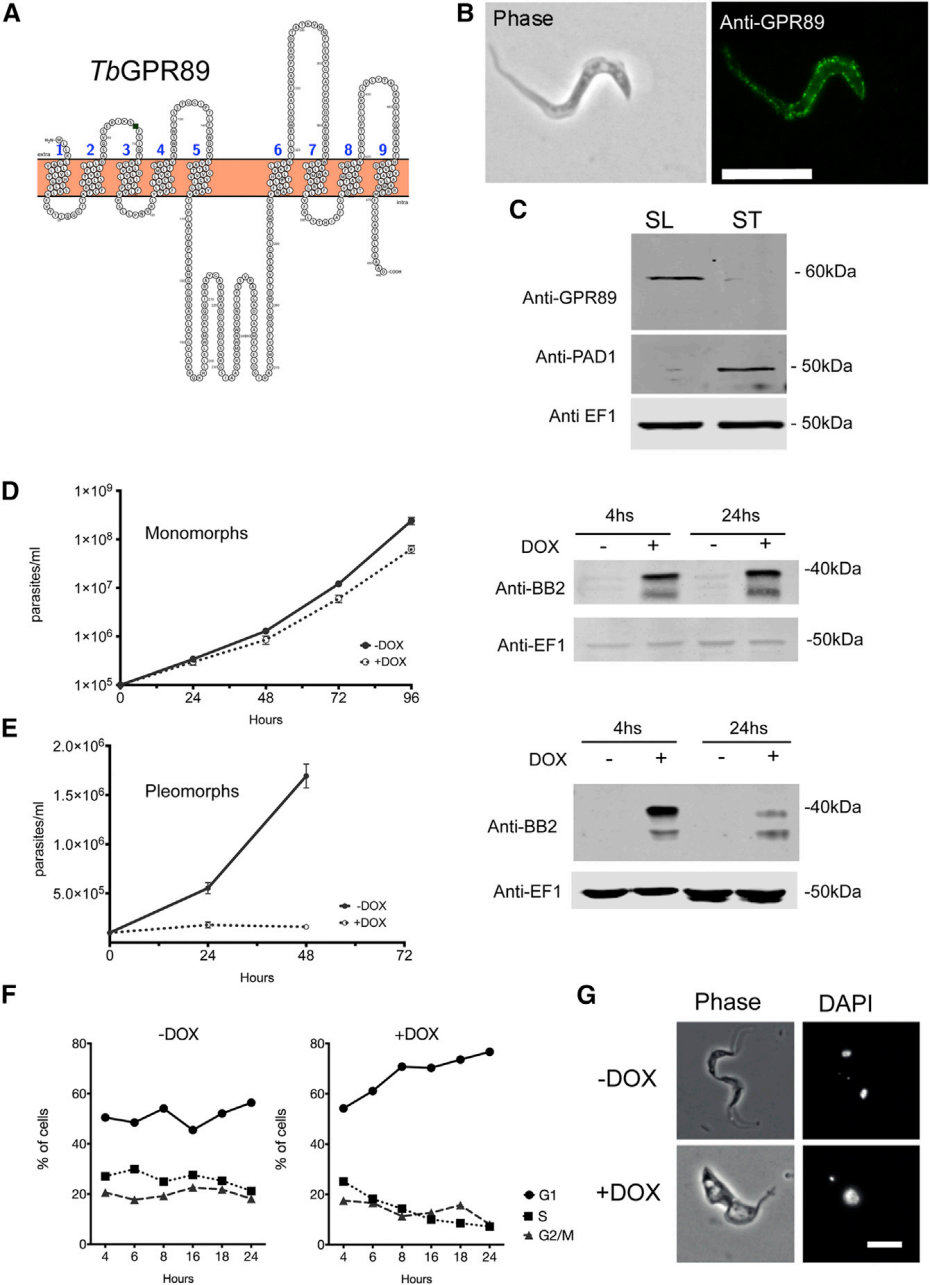
*Tb*927.8.1530 Encodes a GPR89 Family Member that Promotes Stumpy Formation (A) Topology map of *Tb*GPR89 showing the TMDs predicted using the TOPCONs server (http://topcons.cbr.su.se) and rendered via Protter (http://wlab.ethz.ch/protter/start). (B) Location of *Tb*GPR89 on bloodstream form trypanosomes. Left: phase contrast image of a slender bloodstream form trypanosome. Right: surface staining with anti-*Tb*GPR89 antibody. Scale bar, 15 μm. (C) Stage regulation of *Tb*GPR89. Proteins were isolated from parasite populations enriched in slender (SL) forms or stumpy (ST) forms. Samples were reacted with antibodies recognizing *Tb*GPR89, the stumpy specific marker PAD1 or EF1α, as a loading control. *Tb*GPR89 runs aberrantly with respect to its anticipated molecular weight (53 kDa), similar to other GPR89 proteins, likely due to its 9 TMDs and potential post translational modification. (D) Growth of monomorphic Lister 427 90:13 parasites induced (+DOX) or not (−DOX) to express *Tb*GPR89-Ty. Error bars, SEM. Right: protein expression of *Tb*GPR89-Ty1 in monomorphic parasites 4 hr and 24 hr post induction with doxycycline, detected using the Ty1 epitope-specific BB2 antibody. Note that ectopically expressed *Tb*GPR89 predominantly migrates at <40 kDa perhaps due to the efficiency of post translational modification and presence of the epitope tag. Anti EF1α provides the loading control. (E) Growth of pleomorphic *T. brucei* parasites induced (+DOX) or not (−DOX) to express *Tb*GPR89-Ty. Error bars, SEM. Right: protein expression of *Tb*GPR89-Ty1 4 hr and 24 hr post induction with doxycycline. Anti EF1α provides the loading control. (F) Cell-cycle status of pleomorphic *T. brucei* induced (+DOX) or not (−DOX) to ectopically express *Tb*GPR89 in culture. The proportion of cells in G1, GS, or G2/M was determined by flow cytometry. (G) Morphology of pleomorphic *T. brucei* cells induced (+DOX) or not (−DOX) to express *Tb*GPR89-Ty1 in culture for 24 hr. DAPI stains the cell nucleus and kinetoplast. Scale bar, 10 μm. See also Figure S1.

Bioinformatic analysis of the trypanosomatid genomes identified genes encoding representatives of the GPR89 family (Figures S1A and S1B). For *Trypanosoma cruzi* TriTrypDB: TcCLB.508547.140, BLASTP detected similarity scores of 1.1e−16 and 2.3e−16 to *A. thaliana* GTG1 and GTG2, respectively, and 4.1e−10 to mammalian GPR89 (GPHR). The syntenic *T. brucei* gene, TriTrypDB: *Tb*927.8.1530, is predicted to encode 9-TMDs (Tsirigos et al., 2015) and a large central loop (http://wlab.ethz.ch/protter/start) (Figure 1A). All trypanosome family GPR89 family members contain a 70 amino acids GPHR_N (PFAM12537) domain with a conserved LSG motif in the N-terminal 5TM region of mammalian GPHR (http://smart.embl-heidelberg.de). An ABA-GPCR domain (PFAM12430, associated with abscisic acid binding in GTG1) is also present in most kinetoplastid GPR89 homologs (TriTrypDB: TcCLB.508547.140, E value = 8.5e−16) but is not detected in *Tb*GPR89 of *T. brucei* (Figure S1C).

### *Tb*GPR89 Is a Slender Specific Protein that Induces Stumpy Formation via the SIF Signaling Pathway

An antibody targeting *Tb*GPR89 detected expression on bloodstream slender but not on stumpy forms at the cell surface (Figures 1B and 1C). To explore the function of the protein, we transfected parasites with a plasmid driving the doxycycline-inducible ectopic-expression of *Tb*GPR89. In *T. brucei* Lister 427 90:13 monomorphic cells (Wirtz et al., 1999), which have lost the capacity for stumpy formation through serial passage, the protein was effectively expressed but there was only a subtle effect on cell growth (Figure 1D). However, when the protein was inducibly expressed in developmentally competent pleomorphic trypanosomes, *T. brucei* EATRO 1125 AnTa1.1 90:13, the parasites underwent rapid growth arrest in G1 (Figures 1E and 1F) as the cells became morphologically stumpy (Figure 1G). This represented significantly accelerated differentiation compared to the normal differentiation kinetics of wild-type parasites (i.e., stumpy formation in 24 hr rather than >72 hr). In contrast to monomorphic parasites, the protein expression was transient, being detected 4 hr after induction but reduced at 24 hr (compare Figure 1D and 1E), consistent with the developmental loss of the protein in stumpy forms.

**Figure 2:**
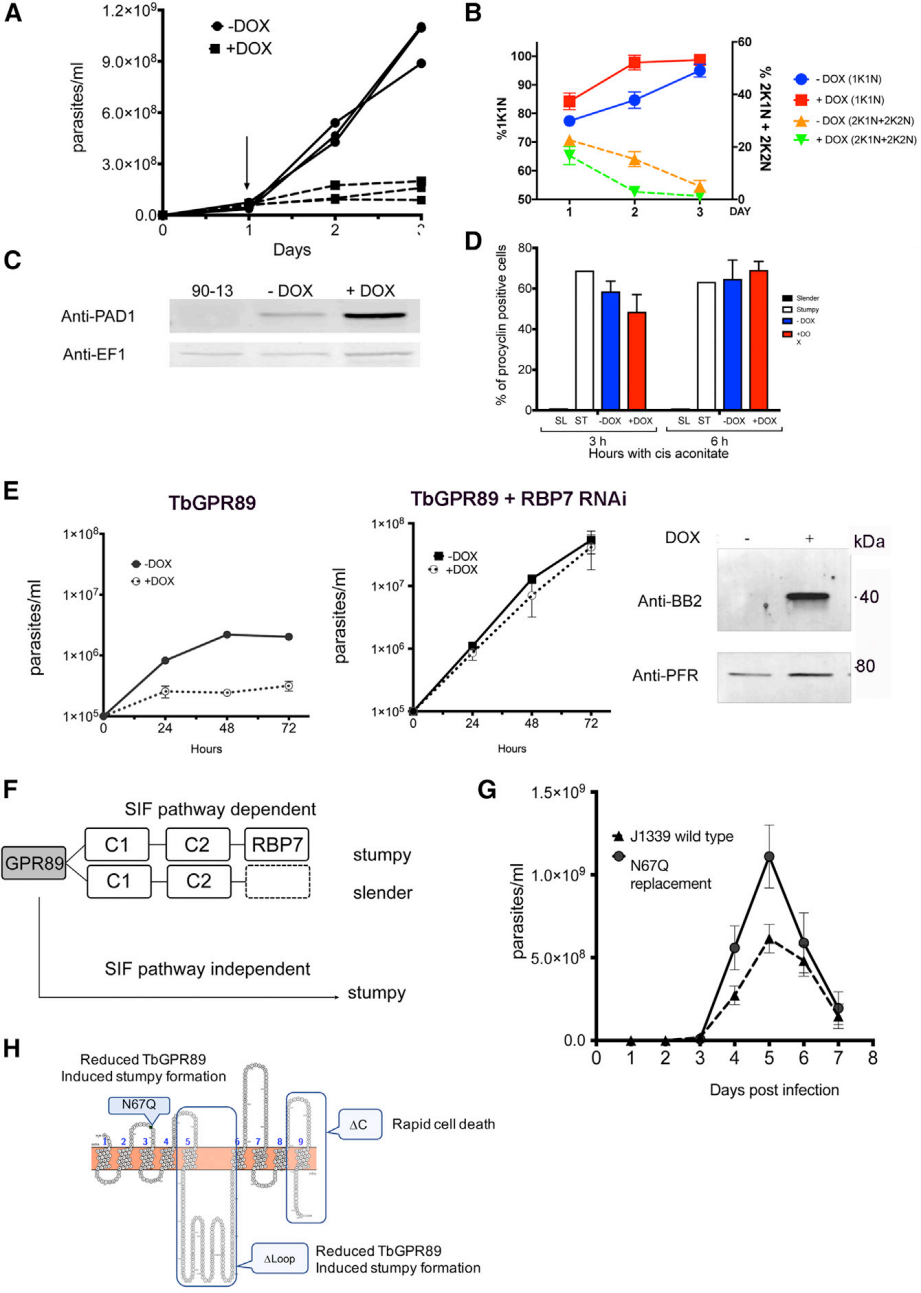
*Tb*GPR89 Expression Drives Stumpy Formation through the SIF Signaling Pathway (A) Parasitemia of pleomorphic *T. brucei* parasites induced (+DOX) or not (−DOX) to ectopically express *Tb*GPR89 *in vivo*. *Tb*GPR89 expression was induced 24 hr post infection by doxycycline (arrowed). n = 3 per group. (B) The percentage of cells with 1K1N or 2K1N plus 2K2N on days 1–3 post infection in the presence or absence of *Tb*GPR89 ectopic expression. n = 3; 250 cells per time point. Error bars, SEM. (C) Expression of the stumpy marker PAD1 is elevated when *Tb*GPR89 expression is induced. Slender parental *T. brucei* EATRO 1125 AnTat1.1. 90:13 (“90-13”) provides the negative control. (D) Expression of EP procyclin on parasites harvested from bloodstream infections and exposed to the differentiation signal, 6 mM *cis*-aconitate. The stumpy form parasites induced to express *Tb*GPR89 (red bars) differentiated as efficiently to procyclic forms as uninduced stumpy forms (blue bars), despite being arrested at lower parasitemia. Independent slender (black bars) and stumpy forms (white bars) provide negative and positive controls, respectively. Error bars, SEM. (E) *Tb*GPR89 expression arrests growth of pleomorphic trypanosomes grown *in vitro* (n = 3) but does not arrest growth when RBP7 expression is silenced by RNAi (n = 3). Error bars, SEM. Uninduced and induced RBP7 RNAi lines were passaged every 24 hr to show that cells continue to proliferate in the presence of *Tb*GPR89 overexpression, as with monomorphic cells. Right: *Tb*GPR89-Ty1 expression in the RBP7 RNAi cells; anti-paraflagellar rod protein is used as a loading control. (F) Representation of the stumpy formation pathway. Components of the SIF-dependent pathway (C1, C2) also include identified molecules such as RBP7, whose silencing inactivates the pathway (Mony et al., 2014). Hence, if *Tb*GPR89-induced stumpy formation is inhibited by RBP7 RNAi, signaling via the SIF pathway is indicated. If not, SIF-independent signaling pathway is implicated. (G) Parasitemia of pleomorphic parental cells and the *Tb*GPR89 WT/N67Q mutants generated by CRISPR. Results from two independent mutant cell lines are shown, both exhibiting elevated parasitemia and delayed differentiation compared to the parent line. Error bars, SEM. (H) Summary of phenotypes generated upon ectopic expression of *Tb*GPR89 mutants detailed in Figure S3. See also Figures S2 and S3.

To establish the physiological relevance of the *Tb*GPR89-induced arrest in pleomorphic parasites, trypanosomes were grown *in vivo*. After 24 hr induction, the parasites underwent rapid growth arrest (Figure 2A), accumulating in a 1K1N cell-cycle configuration (Figure 2B) despite their low parasitemia compared to uninduced parasites. Induced parasites expressed the stumpy-specific cell marker PAD1 (Dean et al., 2009) at day 3 post infection (Figure 2C) and, when exposed to 6 mM *cis*-aconitate, these cells expressed EP procyclin on 69% of cells after 6 hr, reflecting effective differentiation to the next life cycle stage, procyclic forms. This was equivalent to the stumpy forms generated at high parasitemia in uninduced parasites (Figure 2D). Thus, *Tb*GPR89 ectopic expression activates all of the hallmarks of stumpy forms.

Physiological stumpy formation is signaled through a soluble parasite-released “stumpy induction factor” (SIF) (Vassella et al., 1997), whose intracellular signaling pathway has been recently characterized (Mony et al., 2014). To establish whether the *Tb*GPR89-mediated differentiation response was enacted through this pathway, *Tb*GPR89 ectopic expression was induced in cell lines where RBP7, a predicted RNA binding protein necessary for SIF-induced stumpy formation (Mony et al., 2014), was simultaneously and constitutively silenced by RNAi. Figure 2E shows that inducible *Tb*GPR89 ectopic-expression caused growth arrest *in vitro*, whereas simultaneous RBP7 RNAi prevented this, allowing the parasites to continue to grow unimpeded despite effective expression of *Tb*GPR89. This demonstrated that *Tb*GPR89-induced arrest is transduced through the SIF response pathway (Figure 2F).

### *Tb*GPR89 Is an Essential Protein

**Figure S2:**
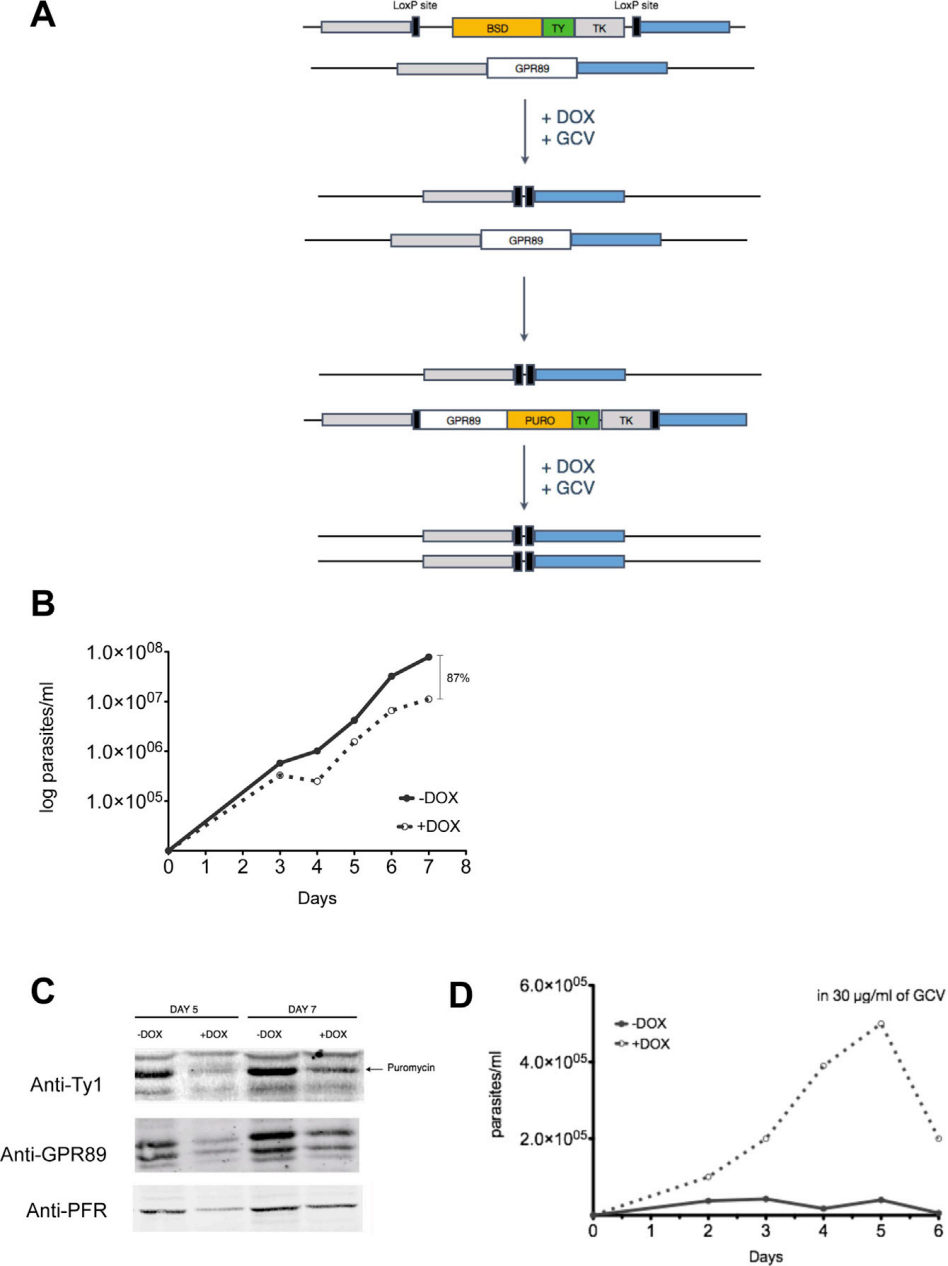
Cre-lox Strategy for the Deletion of *Tb*GPR89, Related to Figure 2 (A–D) For cre-lox based gene deletion, the *Tb*GPR89 gene locus was initially disrupted by integration of a BSD gene and thymidine kinase (TK) flanked by LoxP sites in a cell background capable of doxycycline inducible cre recombinase expression (A). After recombinase induction (+DOX) and ganciclovir (GCV) selection to derive single allele replacements, the cells were transfected with a further loxP-flanked cassette with a *Tb*GPR89 gene linked to a puromycin (PURO) resistance cassette and thymidine kinase (TK), and selectant lines isolated based on their puromycin resistance. Upon doxycycline-mediated cre recombinase induction (+DOX, B) the cells grew more slowly and the expression of both *Tb*GPR89 and puromycin was reduced, indicating deletion of the LoxP flanked cassette in some cells in the population (C). However, when null mutants were selected by cre recombinase induction in the presence of ganciclovir (GCV), the population initially grew but then died after 5 days, when *Tb*GPR89 protein was lost by division (D). Uninduced cells were killed by Gancyclovir through their TK expression.

Attempts to deplete *Tb*GPR89 by RNAi were unsuccessful, as was allelic replacement using drug resistance cassettes targeted to replace both *Tb*GPR89 alleles in the trypanosome’s diploid genome. We also exploited a CreLox/thymidine kinase-based gene deletion system (Kim et al., 2013) to inducibly delete both *Tb*GPR89 alleles (Figure S2A). Cre recombinase induction in combination with ganciclovir selection of null mutants resulted in cell death over 5 days (Figures S2A–S2D). Thus, *Tb*GPR89 is essential in bloodstream slender form parasites.

**Figure S3:**
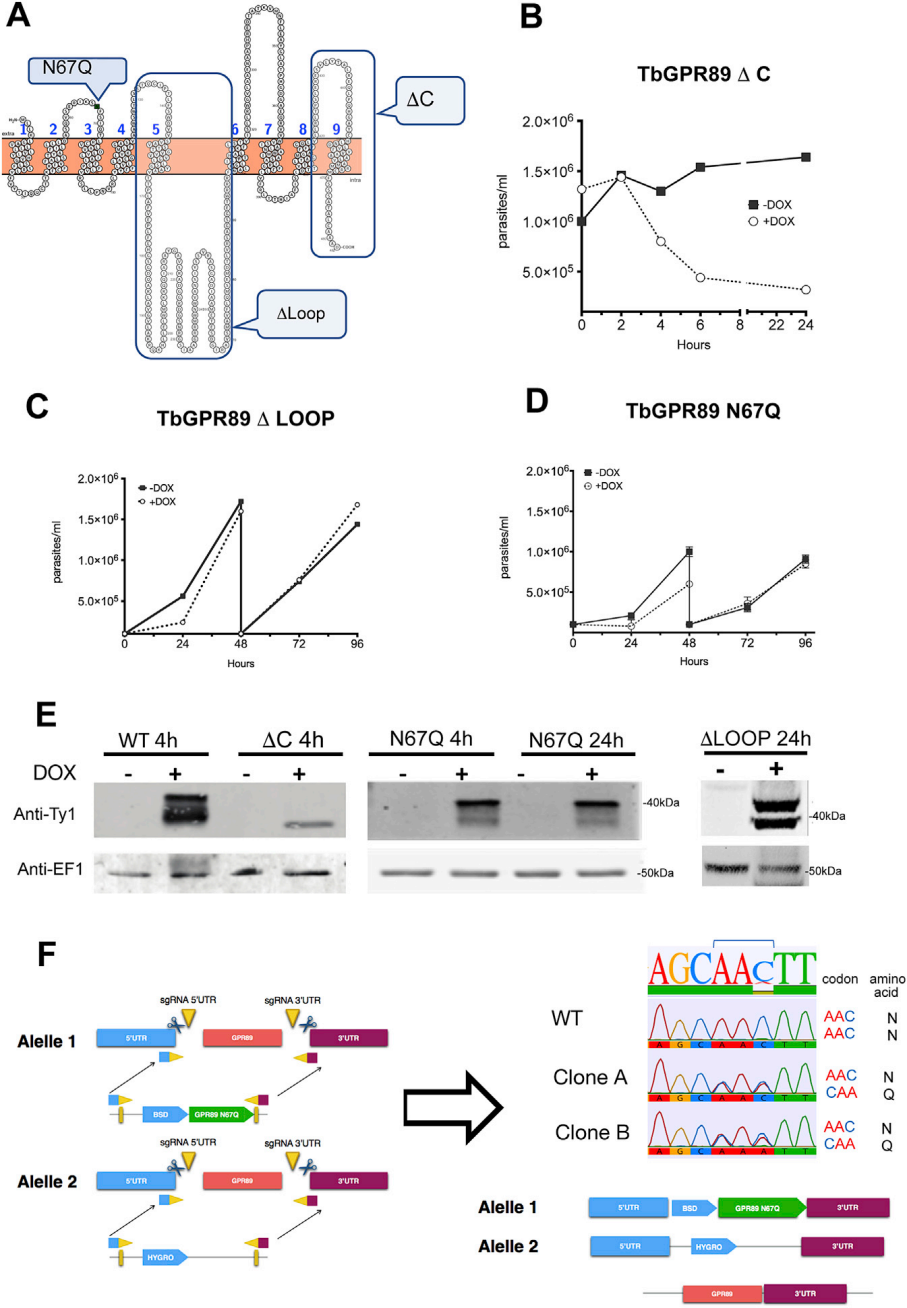
GPR89 Mutants Do Not Drive Stumpy Formation, Related to Figure 2 (A) Schematic representation of different domains mutated within *Tb*GPR89. (B–D) Growth of pleomorphic parasites induced or not to express *Tb*GPR89 with a C-terminal truncation (*Tb*GPR89 ΔC, B), a deleted loop region (*Tb*GPR89 Δ loop; C), or a mutated predicted N-glycosylation site (*Tb*GPR89 N67Q ; D). In C and D, cultures were diluted at 48h to keep cell numbers below 2x10^6^/ml. Error bars = SEM. (E) protein expression of *Tb*GPR89 mutants in the respective cells lines in panels B-D at 4h post induction and, for the N glycosylation site, at 4h and 24h. In each case, the loading control is EF1α. The detected protein in the *Tb*GPR89 ΔC samples is reduced because of the presence of fewer viable cells after induction of the ectopic protein expression. (F) Allelic replacement of wild-type *Tb*GPR89 with *Tb*GPR89 N67Q by CRISPR. One *Tb*GPR9 allele was replaced with the *Tb*GPR89 N67Q mutant (linked to a blasticidin resistance gene) and the other with a hygromycin resistance gene. Analysis of two resulting clones (Clone A, Clone B) showed retention of a wild-type *Tb*GPR89 gene copy, validated by PCR (not shown) and sequence analysis, where both the mutant and wild-type sequence are detected. PCR using primers targeting flanking sequences demonstrated that the mutant allele and hygromycin resistance cassette integrated at the expected genomic location; the additional wild-type allele genomic location has not been mapped, but retains the endogenous 3′UTR (not shown).

Topology modeling and primary sequence analysis of *Tb*GPR89 predicted a number of potential features: an ∼125 amino acid intracellular loop containing the PFAM12537/DUF3735 GPHR family signature, a C-terminal domain with a predicted SCF ubiquitin ligase binding phospho-degron motif (http://elm.eu.org/), and a possible N-glycosylation motif at N67 (Figure S3A). To investigate the importance of these motifs, we expressed mutant forms of *Tb*GPR89 in which the C-terminal amino acids 415–482, containing the phosphodegron motif, were deleted (ΔC), the intracellular loop was deleted and replaced with a 6xHA tag (Δ Loop), or the potential glycosylation site was mutated (N67Q). Each was then ectopically expressed in pleomorphic trypanosomes with the endogenous *Tb*GPR89 gene intact, and their growth and stumpy formation assessed (Figures S3B–S3E). Expression of the C-terminal truncation mutant resulted in death of the parasites within 3 hr of induction, indicating a dominant-negative effect (Figure S3B). In contrast, when the Δ Loop and N67Q mutants were ectopically expressed, the accelerated stumpy induction phenotype of wild-type *Tb*GPR89 was lost and cells continued to proliferate (Figures S3C and S3D). To assess the N67Q mutant in more detail, we generated a Cas9 expressing *T. brucei* pleomorphic line (*T. brucei* EATRO 1125 AnTat1.1 J1339) and used CRISPR technology to replace the wild-type *Tb*GPR89 alleles with the N67Q mutant gene (allele 1) and a hygromycin resistance gene (allele 2). Independent selected cell lines had integrated the HygR gene and the N67Q mutant allele but retained an additional wild-type gene copy, supporting the mutant being nonfunctional (Figure S3F). These cells showed increased growth *in vivo* compared to wild-type *Tb*GPR89, reflecting delayed differentiation (Figure 2G).

These results, summarized in Figure 2H, demonstrated that the accelerated differentiation phenotype generated by *Tb*GPR89 ectopic expression was not simply a consequence of perturbation of the trafficking architecture of the cells, but rather a response resulting from the expression of a surface protein whose function was dependent on its sequence integrity. Furthermore, N67Q/WT cell line analysis supported a role for *Tb*GPR89 in physiological SIF reception and stumpy formation.

### TbGPR89 Can Transport Oligopeptides

**Figure 3:**
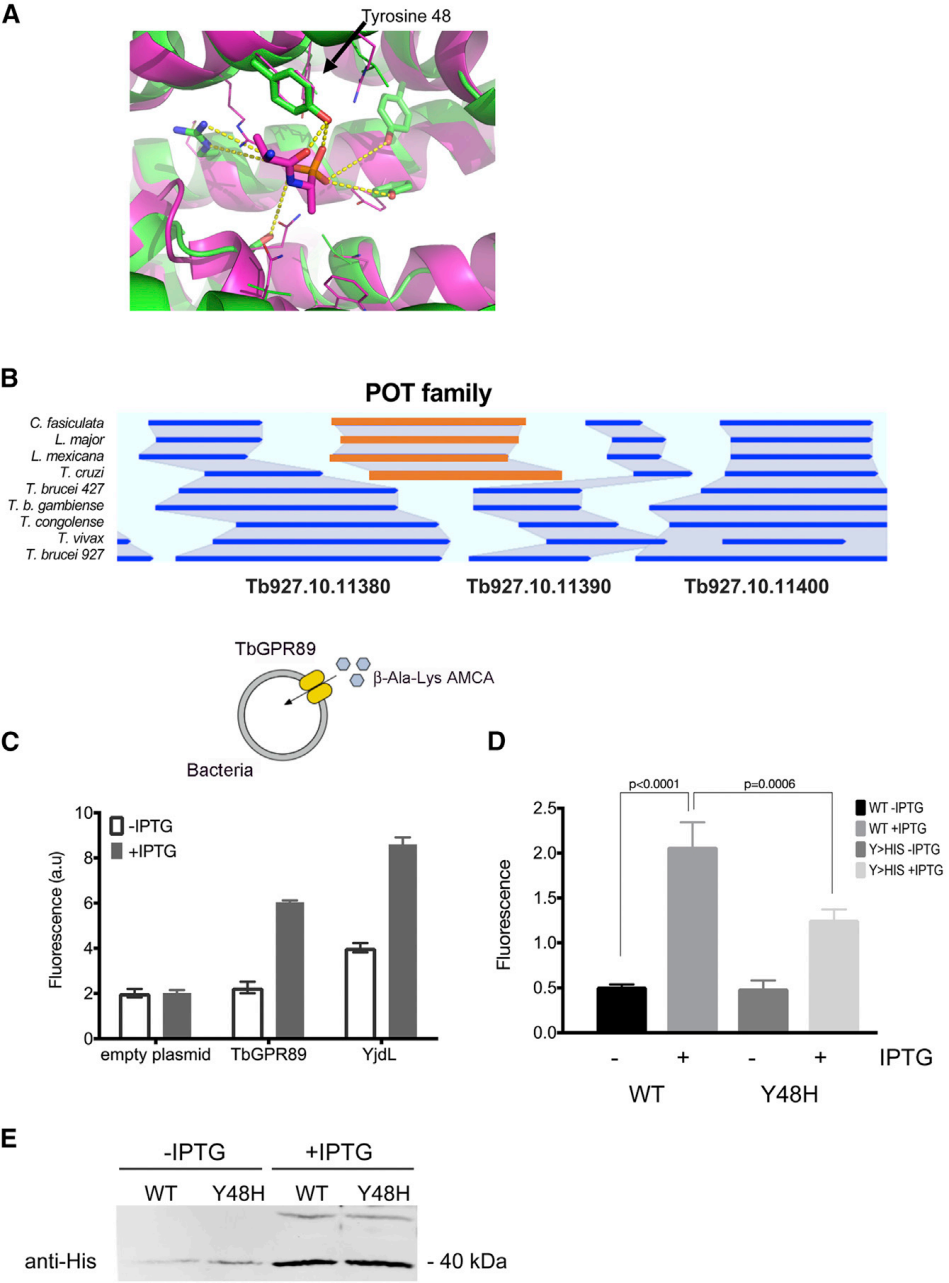
*Tb*GPR89 Transports Oligopeptides (A) Homology modeling of *Tb*GPR89 and the *G. kaustophilus* POT protein. Superimposition of the *Tb*GPR89 model (green) onto the *G. kaustophilus* template (purple), centered on the dipeptide analog alafosfalin binding pocket (residues of which are shown as lines). Side chains of *Tb*GPR89 residues within interaction distance of the ligand are shown as thicker lines. Potential H-bonds between the model and the ligand are highlighted by dashed yellow lines. The predicted substrate interacting tyrosine 48 in *Tb*GPR89 is annotated. (B) Representation of the syntenic regions of the genomes of respective kinetoplastid organisms, with the location of a conventional POT family member highlighted in orange. This is missing in African trypanosomes. (C) Relative uptake of fluorescent dipeptide β-ALA-Lys-AMCA in *E. coli* induced (+IPTG) or not induced (−IPTG) to express *Tb*GPR89, *E. coli* YjdL, or an empty plasmid control. Fluorescence is in arbitrary units. n = 3; error bars, SEM. (D) Mutation of the predicted dipeptide interacting residue tyrosine 48 to histidine 48 in *Tb*GPR89 reduces transport of the fluorescent dipeptide β-Ala-Lys-AMCA when expressed in *E. coli*. Fluorescence is in arbitrary units. n = 3; error bars, SEM. (E) Wild-type and Y48H mutant *Tb*GPR89 are expressed at equivalent levels in induced (+IPTG) and uninduced (−IPTG) *E. coli*. See also Figure S4.

**Figure S4:**
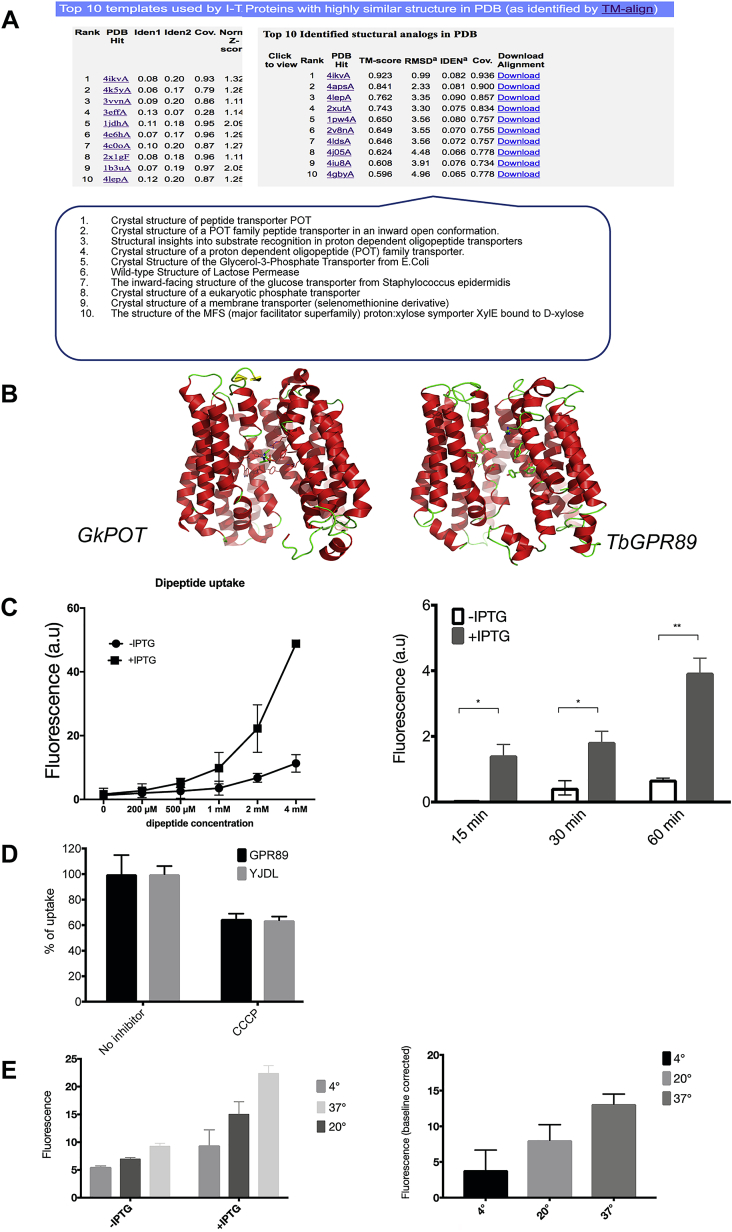
*Tb*GPR89 Is an Oligopeptide Transporter, Related to Figure 3 (A) iTASSER output following submission of the *Tb*GPR89 sequence in September 2015. The description of the top 10 identified structural analogs is shown in the ‘call out’ box. (B) Structural homology between *Tb*GPR89 and structure 4ikvA derived from the *Geobacillus kaustophilus* POT oligopeptide transporter. The *G. kaustophilus* template (PDB: 4IKZ) is shown as secondary structure and colored accordingly, with side chains of the residues of the alafosfalin binding pocket shown as lines and the ligand as sticks. The *Tb*GPR89 model is shown on the right, with equivalent ligand binding pocket residues shown as sticks. Note that the overall model misrepresents the intracellular domain of *Tb*GPR89 as an additional 5TMs because the threading forces a match to the 14 TMs in the *Geobacillus* POT (and other threaded transporters identified by iTASSER). (C) Expression of *Tb*GPR89 generates time and concentration dependent dipeptide uptake when expressed in *E. coli. E. coli* were induced to express *Tb*GPR89 under IPTG induction and monitored for their uptake of β-Ala-Lys-AMCA (measured in arbitrary fluorescence units). The left panel shows uptake of fluorescent dipeptide by *Tb*GPR89 when expressed in *E. coli* is not saturable up to 4mM, consistent with transport but not binding. +IPTG, *Tb*GPR89 expression induced; -IPTG, *Tb*GPR89 expression not induced; the right panel shows the uptake of 200μM β-Ala-Lys-AMCA at 15 min, 30 min and 60min after addition. Error bars = SEM. ^∗^p = 0.006; ^∗∗^p ≤ 0.0001. (D) Inhibition of florescent dipeptide uptake by *Tb*GPR89 or *E. coli* YjdL in the presence of CCCP which inhibits proton gradient-dependent transport. (E) Fluorescent dipeptide uptake by *Tb*GPR89 at 4°C, 20°C or 37°C over 15 minutes. Dipeptide uptake is enhanced at 37°C with respect to 4°C and 20°C, reflective of uptake rather than binding.

Assignment of *Tb*GPR89 to the GPR89 family of proteins was based upon its overall BLAST similarity and conservation of the PFAM12537 domain. To explore tertiary conservation with this or other protein families, *Tb*GPR89 was subjected to structural homology modeling via iTASSER (iterative threading assembly refinement) (Roy et al., 2010) previously used to investigate predicted *Arabidopsis* GPCR proteins (Taddese et al., 2014). Surprisingly, searches revealed structural similarity to voltage-gated ion channels and the POT family of proton-coupled oligopeptide transporters in the substrate recognition region (Figures 3A, S4A, and S4B). POT family transporters are present in a wide range of prokaryotes and eukaryotes and are linked to small molecule uptake. However, a conventional POT gene is missing in African trypanosomes (*T. brucei*, *T. congolense*, *T. vivax*) but not other kinetoplastid species (Figure 3B) leading us to hypothesize that *Tb*GPR89 may replace POT function in these parasites. Therefore, we expressed *Tb*GPR89 in *E. coli* under IPTG-inducible control and monitored the uptake of the fluorescent dipeptide β-Ala-Lys-AMCA compared to the well-characterized *E. coli* POT, YjdL (Ernst et al., 2009). Figure 3C shows uptake of the dipeptidomimetic in *E. coli* that inducibly express *Tb*GPR89. Supporting a transport function for *Tb*GPR89, uptake was non-saturable up to 4 mM, increased over time, and was reduced by the proton-dependent transport inhibitor, carbonyl cyanide m-chlorophenyl hydrazone and at 4°C (Figures S4C–S4E).

Examination of the potential substrate interacting region in *Tb*GPR89 and *Geobacillus kaustophilus* POT, centered on the binding pocket of the dipeptide analog, alafosfalin (Doki et al., 2013) positioned tyrosine 48 in *Tb*GPR89 at a corresponding location to tyrosine 78 in the peptide-binding site of *G. kaustophilus* POT (Figure 3A). When *Tb*GPR89 tyrosine 48 was mutated to histidine (Y48H mutant) and tested for β-Ala-Lys-AMCA transport capability in *E. coli*, uptake was reduced ∼40% (Figure 3D) despite equivalent expression of the wild-type and mutant protein (Figure 3E). This supported the oligopeptide transport function of *Tb*GPR89.

**Figure 4:**
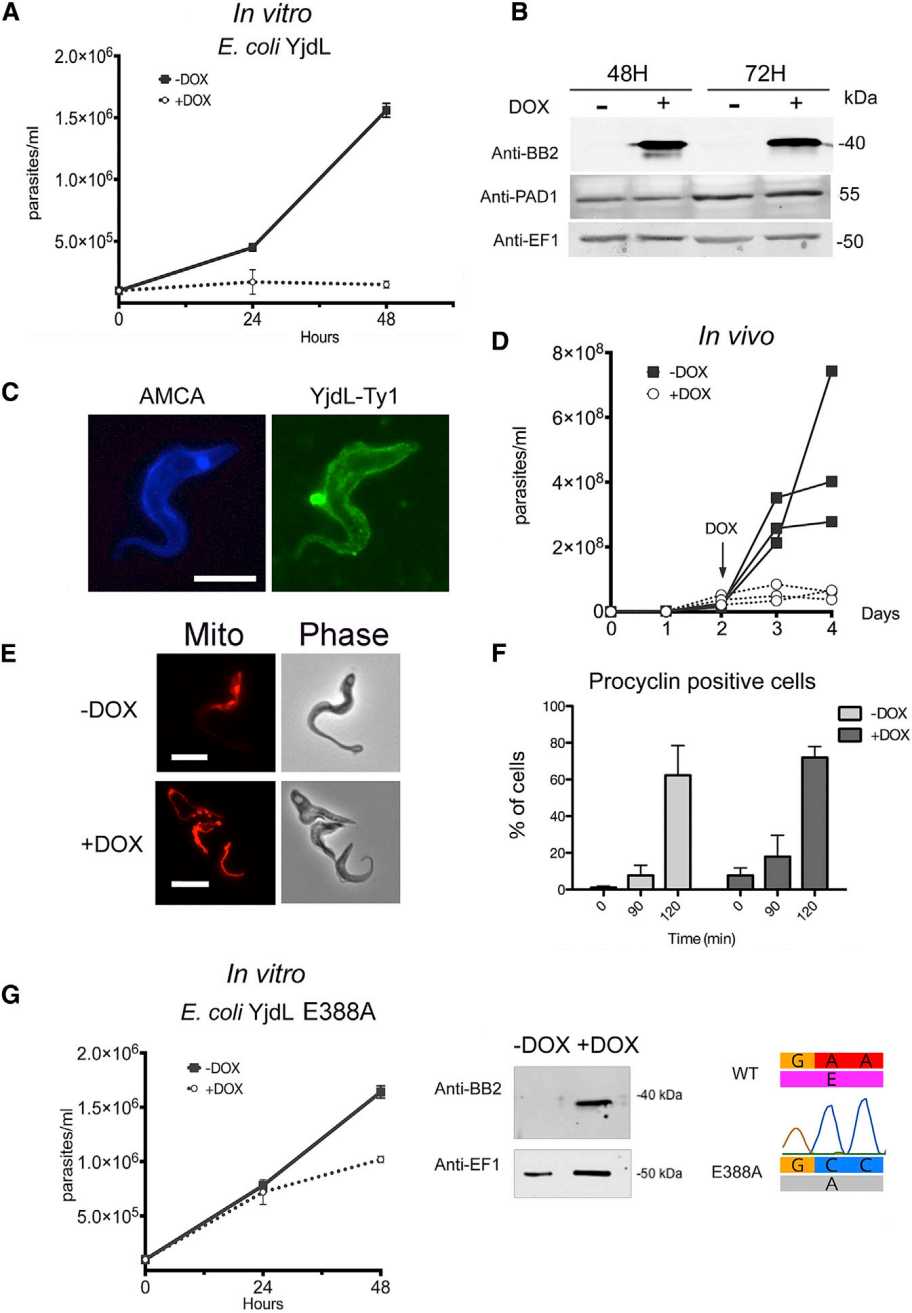
Expression of a Bacterial POT in Trypanosomes Induces Stumpy Formation (A) Expression of *E. coli* YjdL arrests growth of pleomorphic *T. brucei in vitro* when induced with doxycycline. n = 3 per group; error bars, SEM. (B) Expression of *E. coli* YjdL assessed by western blotting 48 hr and 72 hr after induction with doxycycline. PAD1 indicates stumpy formation, evident in the high density uninduced samples and the low density induced samples. Anti-EF1α provides a loading control. (C) *E. coli* YjdL (green) is located on the trypanosome cell surface when expression is induced. AMCA-sulfo-NHS (in blue) labels the parasite surface and flagellar pocket. Scale bar, 10 μm. (D) Expression of *E. coli* YjdL arrests growth of pleomorphic *T. brucei in vivo* when induced with doxycycline. n = 3 +DOX, n = 3 –DOX. (E) Mitochondrial and cell morphology of pleomorphic *T. brucei* induced or not to express *E. coli* YjdL at day 3 of infection. Uninduced cells have a linear mitochondrion (revealed by MitoTracker staining, “Mito”) characteristic of slender forms whereas induced parasites show branched mitochondrial staining and stumpy form morphology. Scale bar, 10 μm. (F) Differentiation to procyclic forms of pleomorphic *T. brucei* after harvest from infection on day 4 and exposure to 6 mM *cis*-aconitate for parasites induced (+DOX) or not (−DOX) to express *E. coli* YjdL. The low parasitemia *E. coli* YjdL-induced stumpy cells, and the uninduced high parasitemia stumpy cells generated by QS differentiated with similar efficiency. Error bars, SEM. (G) Expression of an *E. coli* YjdL E388A mutant in pleomorphic *T. brucei* grown *in vivo*. Reduced differentiation is observed compared to expression of the wild-type YjdL (A) despite effective protein expression (right). The introduced mutation is shown far right. Error bars, SEM.

Having demonstrated that *Tb*GPR89 has oligopeptide transporter activity, we explored whether a heterologous oligopeptide transporter expressed in trypanosomes could promote stumpy formation. Therefore, we expressed Ty1 epitope-tagged *E. coli* YjdL in pleomorphic trypanosomes under doxycycline-regulated control and observed growth arrest *in vitro* within 24 hr (Figure 4A). In this case, protein expression was retained over 72 hr, rather than being lost beyond 24 hr as in *Tb*GPR89 ectopic expression (compare Figures 4B and 1E), presumably due to absence of the phospho-degron domain in the heterologous protein. Furthermore, the YjdL protein was detected at the cell surface (Figure 4C). Induction of *E. coli* YjdL expression also induced rapid growth arrest *in vivo* (Figure 4D) and the generation of morphological stumpy forms that had a characteristic branched mitochondrion (Figure 4E) and were competent for differentiation to procyclic forms (Figure 4F). Supporting a role for oligopeptide transport in the parasite response, ectopic expression of an E388A mutant of YjdL with reduced transport capability (Ernst et al., 2009) resulted in reduced growth arrest and stumpy formation compared with intact YjdL (Figure 4G).

### Oligopeptides Promote Stumpy Formation *In Vitro*

**Figure 5:**
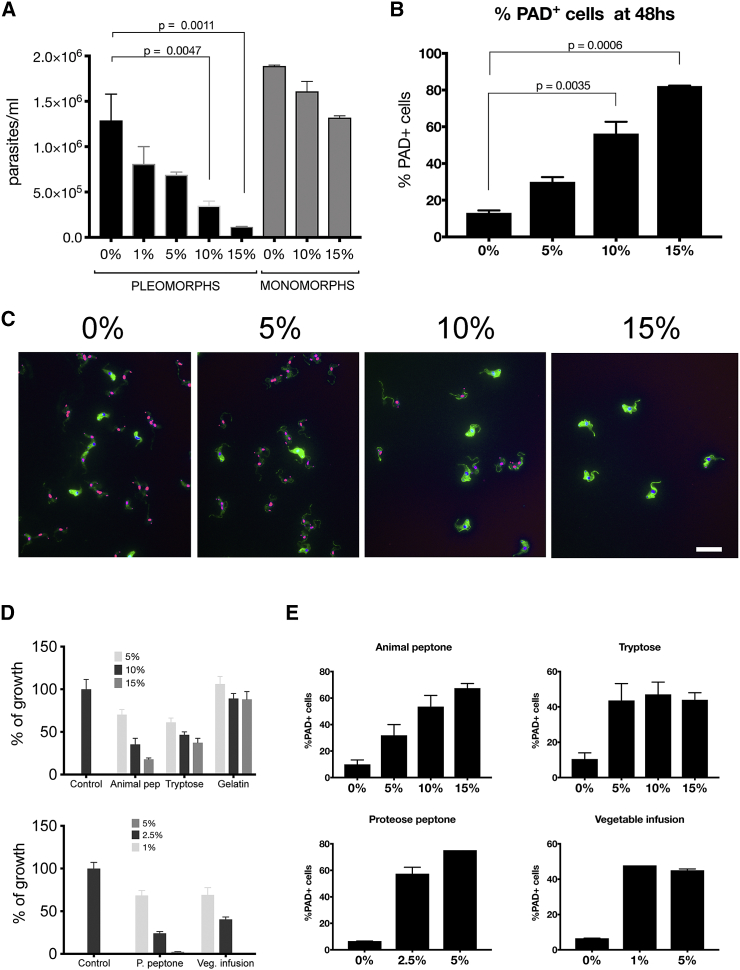
Oligopeptide Mixtures Promote Stumpy Formation *In Vitro* (A) Growth of pleomorphic or monomorphic *T. brucei* cells in varying concentrations of autoclaved brain heart infusion broth at 48 hr. Error bars, SEM. (B) PAD1 expression of pleomorphic *T. brucei* cells in varying concentrations of autoclaved brain heart infusion broth at 48h. Error bars, SEM. (C) Representative images of PAD1 expression and morphology of pleomorphic cells in varying concentrations of BHI broth at 48 hr. PAD1 expression (in green) is evident on increasing proportions of the parasites with higher concentrations of autoclaved BHI; these cells also appear stumpy in morphology. The parasite nucleus and kinetoplast (stained with DAPI) is pseudo colored in magenta. Bar, 25 μm. (D) Growth of pleomorphic *T. brucei in vitro* in the presence of different oligopeptide containing extracts expressed relative to their growth without extract (“control”) at 48 hr. Error bars, SEM. (E) PAD1 expression of pleomorphic *T. brucei* exposed to the different concentrations of oligopeptide containing extracts at 48 hr. Error bars, SEM.

The demonstration that the ectopic expression of both *Tb*GPR89 and a bacterial oligopeptide transporter in trypanosomes promote stumpy formation prompted us to explore the contribution of oligopeptide signaling to differentiation. First, pleomorphic parasites were grown *in vitro* in the presence of 0% to 15% brain-heart infusion (BHI) broth as a source of oligopeptides and the effects on cell proliferation (Figure 5A) and PAD1 expression (Figures 5B and 5C) assayed. In pleomorphic parasites, stumpy formation was observed in a concentration-dependent manner; at 48 hr, cells in 0% and 15% BHI supplement were 15% and 82% PAD1+ve, respectively. Monomorphic cells, which are unresponsive to SIF, showed less effect on cell growth under the same conditions (Figure 5A). Other sources of oligopeptide mixtures also provoked parasite arrest and PAD1 expression in culture, including animal peptone, tryptose, proteose peptone, and vegetable infusion (Figures 5D and 5E).

**Figure 6:**
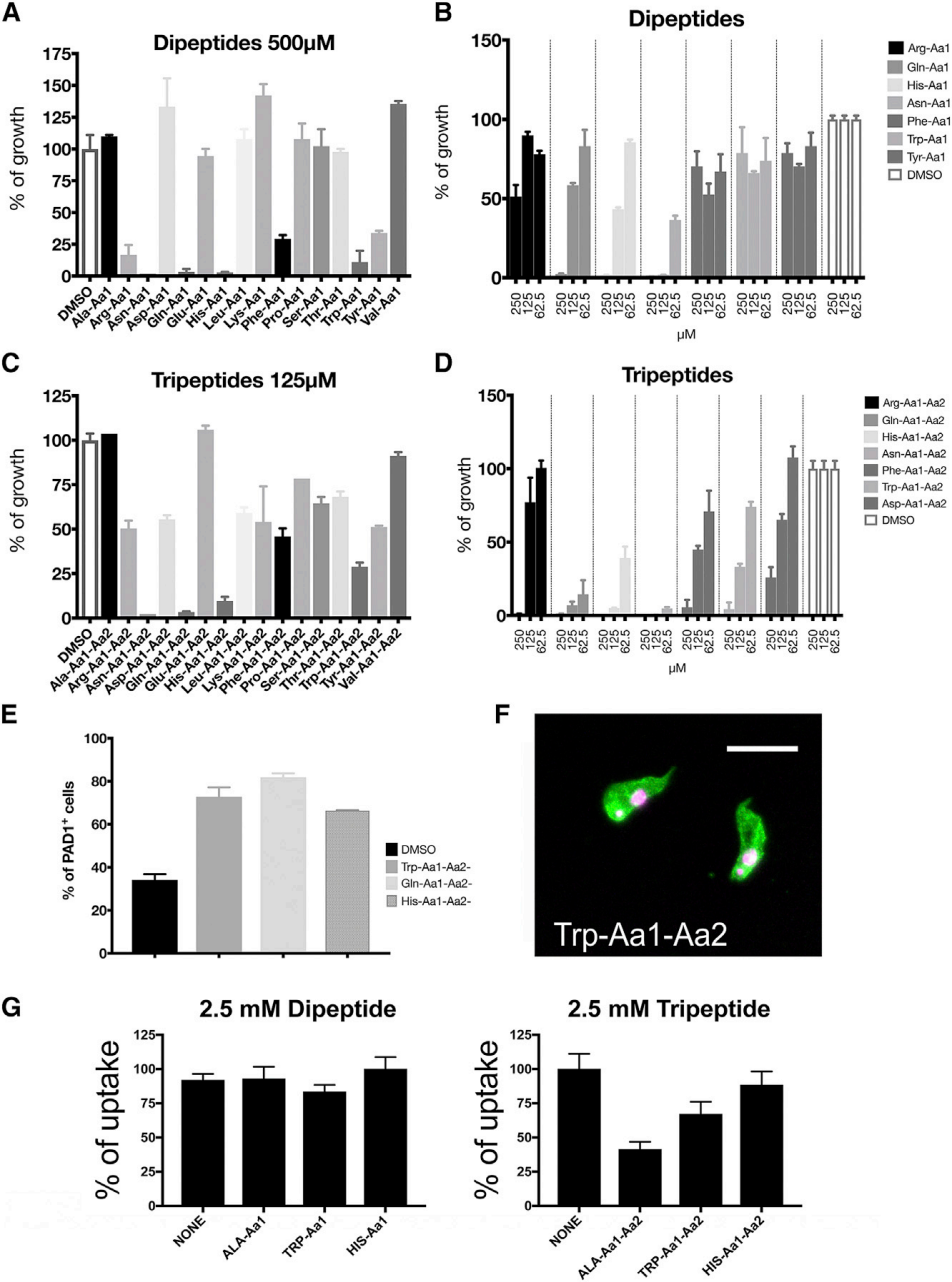
Pleomorphic Trypanosomes Exposed to Dipeptide or Tripeptide Combinations Terminated in Specific N-Terminal Amino Acids (A) The growth of pleomorphic *T. brucei* exposed to 500 μM dipeptide sublibraries over 72 hr compared to DMSO. Error bars, SEM. (B) The growth of pleomorphic *T. brucei* exposed to dipeptide sublibrary titrations from 250–62.5 μM over 72 hr compared to DMSO. Error bars, SEM. (C) The growth of pleomorphic *T. brucei* exposed to 125 μM tripeptide sublibraries (at 500 μM, all tripeptide sublibraries inhibited growth) over 72 hr compared to DMSO. Error bars, SEM. (D) The growth of pleomorphic *T. brucei* exposed to tripeptide sublibrary titrations from 250–62.5 μM over 72 hr compared to DMSO. Error bars, SEM. (E) PAD1 expression by pleomorphic *T. brucei* exposed to 125 μM of the specified tripeptide sublibraries at 72 hr. Error bars, SEM. (F) Immunofluorescence image of PAD1 expression by parasites exposed to 125 μM of the Trp-Aa1-Aa2 sublibrary. Scale bar, 20 μm. (G) The uptake of β-ALA-Lys-AMCA by *E. coli* expressing *Tb*GPR89 in the presence of 2.5 mM competing unlabeled tri- or dipeptide sublibrary. Error bars, SEM.

To explore oligopeptide-mediated stumpy formation in more detail, we chemically synthesized libraries of dipeptides and tripeptides and tested their ability to accelerate stumpy formation. Specifically, combinatorial sublibraries of dipeptides or tripeptides differentiated by N-terminal specific amino acids (i.e., 16 AA-X dipeptides per sublibrary or 256 AA-X-X tripeptides per sublibrary) were evaluated, revealing specificity in the response (Figures 6A–6D). Tripeptides were more potent than dipeptides, but the tripeptides with Asn, Gln, His, Phe, Asp, and Trp at the N terminus were most effective (Figures 6C and 6D). These each arrested growth of the parasites within 48 hr and resulted in PAD1+ve cells demonstrating their effective generation of stumpy forms (Figures 6E and 6F). Correspondingly, tripeptides competed more effectively than dipeptides for β-ALA-Lys AMCA uptake in *E. coli* expressing *Tb*GPR89 (Figure 6G).

### Extracellular Peptidases Generate a Paracrine Signal that Induces Stumpy Formation

**Figure S5:**
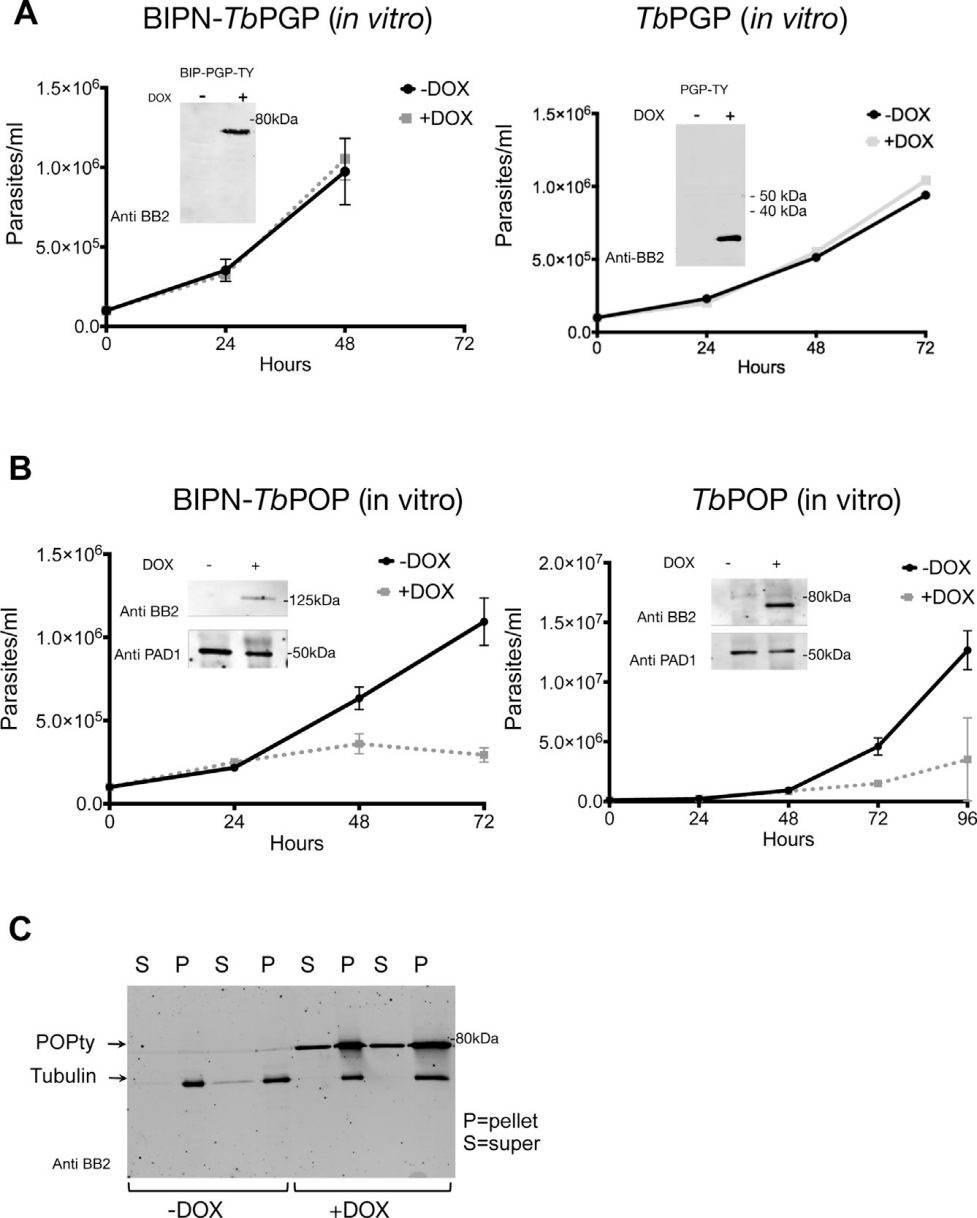
Oligopeptidase Expression in Trypanosomes, Related to Figure 7 (A) Expression *Tb*PGP or BiPN-*Tb*PGP *in vitro*. Panels show the growth of parasites ± induction to express BiPN-*Tb*PGP (left panel) or *Tb*PGP (right panel) *in vitro* (n = 3). In each case expression does not affect the growth of the cells. Error bars = SEM. Inset western blots are shown to confirm protein expression with the BIPN fusion resulting in a larger protein. (B) Expression of BiPN-*Tb*POP or *Tb*POP *in vitro*. The panels show the growth of parasites ± induction to express BiPN-*Tb*POP (left panel) or *Tb*POP (right panel) *in vitro* (n = 3). In each case expression slows the growth of the cells. Error bars = SEM. Inset western blots show the induced expression of *Tb*POP and the expresison of the stumpy marker PAD1, which is present on the high density parasites not induced to express *Tb*POP and the low density parasites induced to express *Tb*POP. (C) Western blot demonstrating the extracellular release of *Tb*POP detected in the culture supernatant (S) with *Tb*POP in the absence of a BIPN secretory signal; samples were prepared after 1h or 2hr incubation in Creek’s minimal medium without serum (Creek et al., 2013) with *Tb*POP expression being induced or not with doxycycline. Tubulin remains in the pellet (P) fraction showing that there is no cell lysis.

**Figure S6:**
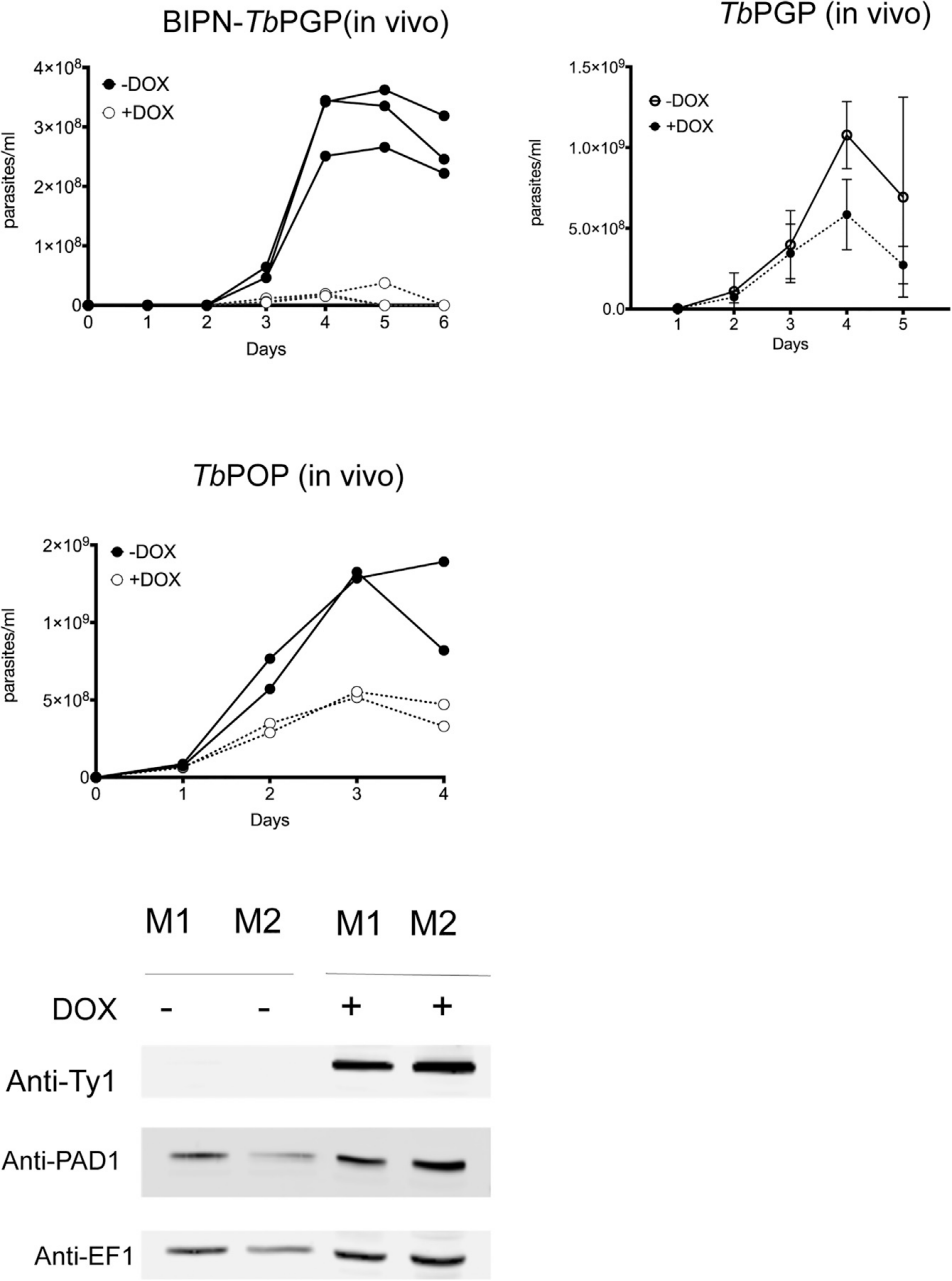
Oligopeptidase Expression *In Vivo* Drives Stumpy Formation, Related to Figure 7 Growth of parasites induced to express BIPN-*Tb*PGP, *Tb*PGP or *Tb*POP *in vivo*. In each case the parasitemias are shown of equivalent numbers of parasites inoculated to initiate the infection, doxycycline being provided to the mice from day 1 of infection. In all cases there is reduced growth upon peptidase expression, this being more pronounced and consistent for BIPN-*Tb*PGP than *Tb*PGP. *Tb*POP was only analyzed without a BIPN leader, because the protein is naturally secreted (Figure S5C). Each growth profile represents analysis of duplicate or triplicate infections for each condition. Error bars = SEM. The western blot demonstrates inducible *Tb*POP expression, with the expression of the stumpy marker PAD1 being present on the high density parasites not induced to express *Tb*POP and the low density parasites induced to express *Tb*POP. EF1 alpha provides a loading control. M; mouse.

We next explored the relevance of oligopeptide signals *in vivo* by manipulating their generation during infections. Trypanosomes release serum-stable peptidases *in vivo*, some of which accumulate at high parasitemia and retain activity in blood (Bossard et al., 2013). Examples are type I pyroglutamyl peptidase (*Tb*PGP, TriTrypDB: *Tb*927.4.2670) that acts on serum substrates with an N-terminal pyroglutamyl residue (Tables S1 and S2) (Morty et al., 2006) and prolyl oligopeptidase (*Tb*POP; TriTrypDB: *Tb*927.10.8020), which cleaves after proline residues (Bastos et al., 2010) (Tables S3 and S4). *Tb*PGP is a cytosolic peptidase released by lysed parasites during infections (Morty et al., 2006) whereas *Tb*POP is reported to be secreted (Geiger et al., 2010). To determine if the activity of these trypanosome-derived oligopeptidases in blood could affect stumpy formation, we generated transgenic parasite lines that express *Tb*PGP or *Tb*POP with a C-terminal Ty1 epitope and also modified *Tb*PGP with a BIP N-terminal fusion (BIPN-*Tb*PGP) promoting extracellular secretion (Bangs et al., 1996). *In vitro*, the inducible expression of *Tb*PGP and BIPN-*Tb*PGP did not affect cell growth (Figure S5A), indicating their expression was not deleterious. In contrast, TbPOP expression slowed growth and was detectably secreted (Figures S5B and S5C). Strikingly, however, pleomorphic cells induced to express either oligopeptidase *in vivo* arrested and differentiated to stumpy cells at lower parasitemia than in uninduced cells. Moreover, this effect was more rapid and pronounced in parasites expressing secreted *Tb*PGP fused with a BIPN leader than in parasites expressing the native *Tb*PGP (Figure S6).

**Figure 7:**
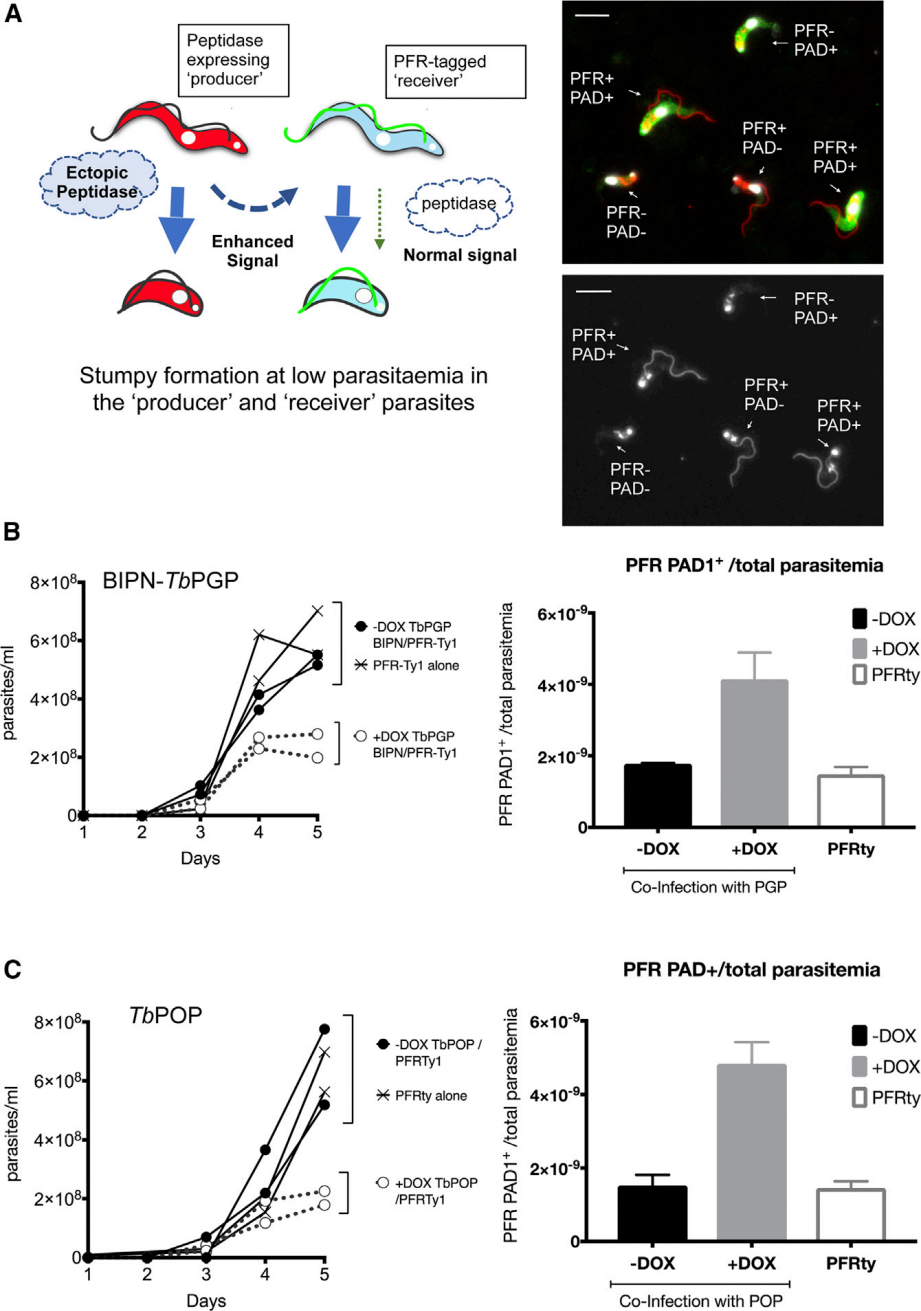
Peptidase-Expressing Bloodstream Trypanosomes Generate a Stumpy-Inducing Paracrine Signal (A) Schematic representation of the experimental regimen. Trypanosomes were induced to express secreted peptidases under doxycycline regulation, so generating an enhanced signal that promotes stumpy formation (“Producer line”). Co-infection with pleomorphic *T. brucei* cells with a Ty1 epitope tagged PFR acts as a “receiver” cell line that can be distinguished from “producer” cells via labeling of the flagellum. Right: representative field comprising “producer” cells (PFR^−^) and “receiver” cells (PFR^+^) co-labeled or not with the stumpy marker, PAD1 (green). Scale bar, 15 μm. (B) Parasitemias of mice infected with the PFR-Ty1 cell line alone, or a coinfection of the PFR-Ty1 cell line with the BIPN-*Tb*PGP line either induced or not to express the peptidase by doxycycline. Right: percentage PAD1^+^ PFRTy1 divided by the overall parasitemia revealing that the PFR-TY1 cells are induced to become stumpy despite the low parasitemia of the coinfection when induced. Data are derived from microscopic analysis of 2,000 cells in each sample on day 5 of infection; for PFR-Ty1 cells, >250 cells were scored as PAD1^+^ or PAD1^−^. Error bars, SEM. (C) Parasitemias of mice infected with the PFR-Ty1 cell line alone, or a coinfection of the PFR-Ty1 cell line with the *Tb*POP line either induced or not to express the peptidase by doxycycline regulation. Right: percentage PAD1^+^ PFRTy1 cells divided by the overall parasitemia revealing that the PFR-TY1 cells are induced to become stumpy despite the low parasitemia of the coinfection when induced. Data are derived from microscopic analysis of 2,000 cells in each sample on day 5 of infection; for PFR-Ty1 cells, >250 cells were scored as PAD1^+^ or PAD1^−^. Error bars, SEM. See also Figures S5, S6, and S7 and Tables S1, S2, S3, and S4.

**Figure S7:**
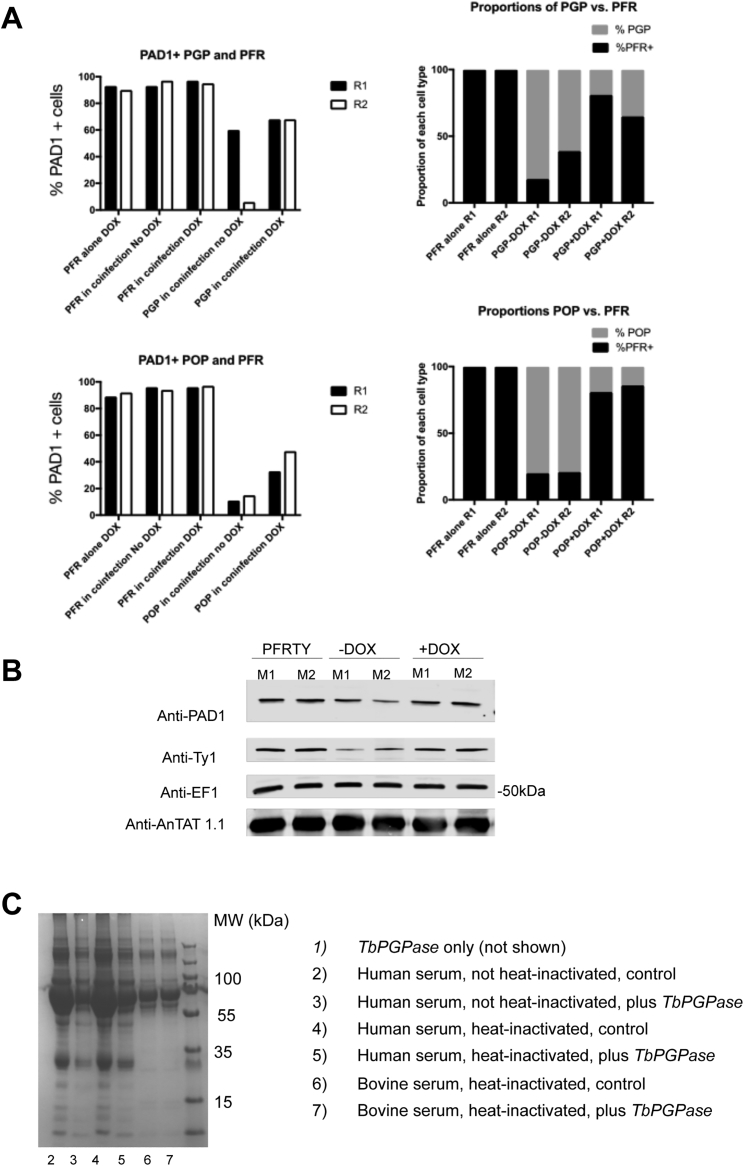
Oligopeptidase Expression Generates a Paracrine Signal Driving Stumpy Formation, Related to Figure 7 (A) Coinfection of BIPN-PGP or *Tb*POP expressing cells (‘Producers’) with PFR-Ty1 pleomorphic parasites (‘receivers’). The histograms represent the % PAD1 cells for each cell type. PFR1-Ty1 cells express high levels of PAD1 at high parasitaemia (either when in a monoinfection or when co-infected with the producer line not induced to express the peptidase). With peptidase induction, the overall parasitemia is much lower (Figure 7) but high levels of PAD1 expression are expressed on the ‘receiver’ PFR-Ty1 line. The relative proportion of producer and receiver cells was also determined by microscopical scoring of the number of parasites with a labeled flagellum after reaction with the Ty1 specific antibody BB2, with at least 2000 cells analyzed in each infection, except the monoinfection with PFR-Ty1 cells alone (where all cells were PFR labeled from an analysis of at least 250 cells). The relative proportion of PFRTy1 cells was higher upon peptidase induction reflecting either reduced growth of the producer cells upon peptidase expression or a combination of autocrine and paracrine induced arrest in the producer cells, while receiver cells only exhibit a paracrine response to the induced peptidase expression. (B) The western blot detects the stumpy marker PAD1, which is present on the high density parasites not induced to express *Tb*POP and the low density parasites induced to express *Tb*POP, as well as the high density PFR-ty tagged cells alone. BB2 detects the epitope tag present on the PFR-Ty1 tagged ‘recipient cell line’, EF1alpha provides a loading control and the AnTat1.1 lanes demonstrate the integrity of the VSG regardless of the expression of the *Tb*POP peptidase by the producer cells. (C) Samples of human and bovine serum were mixed with an equal volume of standard PGPase assay buffer (50 mM HEPES, 1 mM EDTA and 10 mM DTT) and incubated overnight at 37°C with either recombinant *Tb*PGP (5μl per 100μl) or an equivalent volume of assay buffer. When centrifuged following incubation, significant-sized, gelatinous (‘clot-like’) pellets were formed in the human serum samples which had been incubated without *Tb*PGP; these pellets were much smaller in the samples incubated with *Tb*PGP and were virtually absent from the bovine serum samples. The pellets were solubilised in equal volumes of SDS-PAGE loading buffer and analyzed by SDS-PAGE (see below). The pellets appeared to be composed of bulk serum proteins with no obvious enrichment for specific proteins, however, the total amount of protein was clearly much reduced in the human serum samples which included *Tb*PGPase.

We then investigated whether expression of trypanosome oligopeptidases could generate an inter-cellular paracrine signal to promote differentiation in co-infecting parasites. To test this, BIPN-*Tb*PGP or *Tb*POP expressing trypanosome lines were co-infected with a distinct pleomorphic reporter cell line modified to encode a Ty1 epitope-tagged paraflagellar rod protein (Silvester et al., 2017), allowing the visual discrimination of “producer” (peptidase secreting) and “receiver” (PFR-Ty1) cells (Figure 7A). Infections were initiated with PFR-Ty1 cells alone, or with PFR-Ty1 cells in combination with cells induced or not to express each oligopeptidase. The parasitemia of resulting infections and the proportion of “producer” (BIPN-*Tb*PGP or *Tb*POP) and “receiver” (PFR-Ty1) cells was then scored, with co-labeling for PAD1 identifying the proportion of stumpy cells for each cell type (Figure 7A, right). Figures 7B and 7C show that expression of either BIPN-*Tb*PGP or *Tb*POP restricted growth of the total parasite population, which grew to a much lower level than in a single infection or when peptidase expression was not induced in the co-infecting population. Despite the lower overall parasitemia, the “receiver” PFR-Ty1 cells in the oligopeptidase-induced co-infection were highly enriched for stumpy forms (Figures 7B, 7C, and S7A). Moreover, analysis of the relative proportion of “producer” and “receiver” cells in each group demonstrated that the “producer” cells were more affected than the “receiver” cells by the oligopeptidase expression, as their overall levels diminished as a contribution to the total parasitemia (Figure S7A). These observations are all consistent with the hypothesis that secreted oligopeptidases promote stumpy formation as a paracrine response in the “receiver” cells, and the producer cells are affected by their production of the peptidase *in vivo*, or by a combination of a local autocrine and paracrine response. We conclude, therefore, that peptidases released by trypanosomes *in vivo* can generate a paracrine quorum sensing signal to induce stumpy formation.

## Discussion

Our data reveal oligopeptide signaling can promote trypanosome QS consistent with the activity of the proposed stumpy induction factor. This is based upon several key discoveries; (1) a GPR89 family member with oligopeptide transport capability, (2) the surface location of this protein on the parasite stage that receives the QS signal, (3) the ability of ectopically expressed *Tb*GPR89 and a heterologous oligopeptide transporter to drive stumpy formation, (4) the capacity of specific oligopeptides to promote stumpy formation *in vitro*, and (5) the ability of secreted oligopeptidases, a normal part of trypanosome infection, to provide a paracrine signal promoting stumpy production in co-infecting parasites. Collectively, these provide a “signal” and “receptor” mechanism for density sensing in trypanosome infections, processes that have been the subject of speculation for over 20 years.

In both eubacteria and eukaryotes, diverse oligopeptides are transported by promiscuous POT family proteins to provide nutritional carbon or nitrogen sources for cells, these often being generated by extracellular peptidases. POT family proteins are also present in kinetoplastids, except the African trypanosomes (*T. brucei*, *T. congolense*, *T. vivax*), where the POT gene has apparently been lost by gene deletion. These trypanosomes all show density-dependent growth control in the mammalian bloodstream (Shapiro et al., 1984, Silvester et al., 2017, Vassella et al., 1997), this being linked to the development of stumpy forms in *T. brucei*. We show that *Tb*GPR89 is an essential protein in trypanosomes that can replace the oligopeptide transport function of a conventional POT but also provides a density sensing role in trypanosome quorum sensing. This dual function provides an elegant mechanism for signal perception where *Tb*GPR89 enables essential oligopeptide uptake to the proliferating slender forms but also ensures there is a single signal input for QS without interference from additional oligopeptide uptake by a conventional POT. An accelerated developmental response to ectopic overexpression of *Tb*GPR89 may reflect increased sensitivity to the signal, although oligopeptide transporters may also be regulated by oligomerization (Guettou et al., 2013, Newstead, 2015) to precipitate differentiation. The intracellular oligopeptide specificity and response remains to be dissected, but may link to TOR and AMPK, which are regulators of trypanosome differentiation (Barquilla et al., 2012, Saldivia et al., 2016).

Several peptidases are released by trypanosomes that accumulate and are active in the bloodstream during infections (Bossard et al., 2013, Moss et al., 2015). These and additional host peptidases degrade numerous host substrates, to generate diverse oligopeptide signals that may be transported by *Tb*GPR89. Here, we demonstrated that two released peptidases, prolyl oligopeptidase (*Tb*POP) (Bastos et al., 2010) and pyroglutamyl peptidase (*Tb*PGP) (Morty et al., 2006), increase stumpy formation. We show that *Tb*POP is secreted by trypanosomes, consistent with its detection in the excretory/secretory material of the parasite (Geiger et al., 2010). Furthermore, although *Tb*PGP is normally released by lysed trypanosomes, we observed enhanced stumpy formation when this protein is modified to enhance its secretion. That both peptidases generated the paracrine signal suggests, therefore, that common products, generated directly or after further processing in the blood, contribute to the signal.

Oligopeptides are enriched in both trypanosome conditioned medium (Creek et al., 2013) and the serum of infected patients (Vincent et al., 2016). Supporting this, we show that *Tb*PGP and *Tb*POP degrade serum proteins (Figure S7C; Tables S1 and S3), and addition of oligopeptide mixtures to trypanosome media promotes stumpy formation of pleomorphic trypanosomes but not monomorphic parasites that are QS-signal blind. The nature of the defect in these monomorphic cells remains to be discovered but is not at the level of *Tb*GPR89 because this protein is essential in bloodstream form parasites.

Both *Tb*PGP and *Tb*POP exhibit activities that may be relevant for trypanosome QS. Thus, *Tb*PGP degrades pyroglutamyl groups on serum peptides such as thyroid releasing hormone (TRH) or gonadotrophin-releasing hormone (GnRH), an activity we have confirmed in human and bovine serum (Table S1), and many additional *Tb*PGP-generated products were detected that will require extensive further analysis for unambiguous identification (Table S2). *Tb*POP can also act on bioactive peptide hormones as well as abundant host collagen and proline-rich proteins in serum (Bastos et al., 2010) (Tables S3 and S4). Using synthetic di- and tripeptide libraries, we have further established that there is specificity to the oligopeptide signal with selectivity for several tripeptide sublibraries, and some members of these are detected as direct products of the secreted peptidases in serum.

The ability of exogenous oligopeptides and secreted peptidases to induce premature differentiation *in vivo* supports a role for oligopeptides in inter-cellular QS. Moreover, the absence of an effect of *Tb*GPR89 expression in developmentally incompetent parasites (monomorphs and RBP7-depleted cells), and the accelerated differentiation of a co-infecting “receiver” line demonstrates that the developmental response is not a consequence of the action of intracellular peptidase trafficking or activity at the expresser cell surface. Indeed, the expressed VSG remains intact (Figure S7B), arguing against a SIF-independent differentiation response activated by perturbation of the surface coat (Zimmermann et al., 2017). Instead, our data suggest a model where released peptidases act as public goods (Brown and Taddei, 2007) to generate a paracrine oligopeptide signal that can promote differentiation. This is consistent with the reported properties of SIF (<500 Da, heat stable) but differs from the anticipated characteristic of SIF as a directly released metabolite or small molecule (Vassella et al., 1997).

A “stumpy induction factor” signal generated in the environment by the release of parasite proteases is consistent with environmental sensing in other organisms and the biological characteristics of trypanosome infection *in vivo*. For example, a recently reported fungal signaling system is dependent upon the release of extracellular oligopeptidase (Homer et al., 2016), and in *Bacillus cereus*, QS signaling operates by the extracellular processing of the autoinducing peptide by a secreted neutral peptidase B, and then import by an oligopeptide permease (Lazazzera and Grossman, 1998). The local production of peptidases is also compatible with the generation of stumpy forms when parasites are constrained in the host dermis (Capewell et al., 2016) or adipose tissue (Trindade et al., 2016) as well as at high density in the bloodstream circulation of infected mice. This is because both environmental flow and cell density would determine the concentration of oligopeptide signals generated, with tissue-resident parasites in a low flow environment and in close proximity to peptidase substrates (Caljon et al., 2016) predicted to differentiate at lower density than circulating parasites in a high flow blood environment. Such local effects can also explain how livestock trypanosome infections can sustain transmissibility while exhibiting low bloodstream parasitemia. Immune-mediated parasite killing could also boost the generation of transmission stages through peptidase release from dying parasites.

Our results have implications for two potential therapeutic approaches. First, the delivery of a stable oligopeptide signal to promote premature stumpy formation could generate an anti-virulence “quorum-sensing interference” approach if comprehensively and systemically active. Alternatively, our discovery that a GPR89 family protein is required for cell viability and cell-type differentiation provides opportunities for pharmacological intervention. GPCR-like proteins as well as multi-membrane spanning transporters and transceptors are highly targeted in drug discovery programs, with nearly 40% of current drugs focused on this family of proteins. In particular, the functions of *Tb*GPR89 in both slender form viability and parasite stumpy formation provides an evolution-proof double lock to prevent the emergence of drug-resistance, since any viable drug-resistant mutants bypassing *Tb*GPR89 would be unable to spread through their transmission incompetence.

## STAR★Methods

### Key Resources Table

**Table:** 

REAGENT or RESOURCE	SOURCE	IDENTIFIER
**Antibodies**		

Anti *Tb*GPR89	This paper; raised to peptide: LDASQVSERIKSNFS	N/A
Anti-PAD1	Dean et al., 2009	N/A
Anti-EF1 alpha	Merck Millipore	Cat#05-235; RRID:AB_309663
Anti EP procyclin; Clone *TB*RP1/247 ascites	Cedar Lane Laboratories	Cat#CLP001A; RRID:AB_10060662
Anti-Ty1 epitope tag specific BB2 antibody	Bastin et al., 1996; hybridoma cell line a gift of Keith Gull, Oxford University/available through Thermofisher	Cat#MA5-23513; RRID:AB_2610644
Anti-His Tag	Sigma	Cat# SAB4301134
IRDye 680 goat anti-mouse secondary antibody	Li-Cor	Cat#P/N 925-68070; RRID:AB_2651128
IRDye 800CW Goat anti-Mouse IgG (H + L) secondary antibody	Li-Cor	Cat#P/N 925-32210; RRID:AB_2687825
Anti-rabbit (goat anti-rabbit IgG (H+L) Dylight 800	Thermofisher	Cat#SA5-10036; RRID:AB_2556616

**Bacterial and Virus Strains**		

*E. coli* BL21-CodonPlus (DE3)-RIPL	Agilent	Cat#230280

**Chemicals, Peptides, and Recombinant Proteins**		

AMCA; 7-Amino-4-methyl-3-coumarinylacetic acid	Sigma Aldrich	Cat#08445
β-Ala-Lys-AMCA (β-Ala-Lys-Nε-7-amino-4-methyl-coumarin-3-acetic acid)	Fisher Scientific	Cat#NC0906036
4′,6-diamidino-2- phenylindole (DAPI)	Sigma Aldrich	Cat#10236276001
Brain Heart Infusion broth	Sigma Aldrich	Cat#53286
Vegetable Special infusion	Sigma Aldrich	Cat#95757
Proteose peptone (Vegetable)	Sigma Aldrich	Cat#29185
Peptone from animal proteins	Sigma Aldrich	Cat#77180
Peptone from Gelatin	Sigma Aldrich	Cat#70951
Tryptose	Fluka	Cat#70937
HMI-9 Medium	Life Technologies	Cat#074-90915
SDM-79 medium	Life Technologies	Cat#074-90916
Amaxa basic parasite nucleofector kit 2 solution	Lonza	Cat#VMI-1021
Quickchange II site directed mutagenesis kit	Agilent	Cat#200523
Cis aconitate	Sigma	Cat#A3412
Carbonyl cyanide m-chlorophenyl hydrazine (CCCP)	Sigma	Cat#C2759
Ganciclovir	Sigma	Cat#G2536
TLCK (*N*_α_-Tosyl-L-lysine chloromethyl ketone hydrochloride)	Sigma	Cat#T7254

**Critical Commercial Assays**		

Mitotracker Red CMXRos	Thermofisher	Cat#M7512

**Experimental Models: Organisms/Strains**		

*Trypanosoma brucei* EATRO 1125 AnTat1.1 90:13	Engstler and Boshart, 2004	N/A
*Trypanosoma brucei* Lister 427 90:13	Wirtz et al., 1999	N/A
*Trypanosoma brucei* EATRO 1125 AnTat1.1 J1339	This study	N/A
Mouse MF1	Charles River	N/A

**Oligonucleotides**		

Refer to Table S5	N/A	N/A

**Recombinant DNA**		

pDEX577-Y	Kelly et al., 2007	N/A
pyrFE-BSD	Kim et al., 2013	N/A
pyrFE-PUR	Kim et al., 2013	N/A
pET28a	Novagen	Cat#69864
pPOTv6	Kindly provided by Dr Sam Dean, Oxford University	N/A
pPOTv7	Kindly provided by Dr Sam Dean, Oxford University	N/A
pJ1339	Kindly provided by Dr Jack Sunter, Oxford Brookes University	N/A
pLEW100cre-EP1	Kim et al., 2013	N/A
pyrFEKO-BSD	Kim et al., 2013	N/A
pyrFEKO-GPR89ty-BSD	This paper	N/A
pyrFE-PUR-GPR89 UTRs	This paper	N/A
pPOTv6-GPR89-N67Q	This paper	N/A
pDex577-TbGPR89Ty1	This paper	N/A
pDex577-TbGPR89-N67QTy1	This paper	N/A
pDex577-TbGPR89-ΔCTy1	This paper	N/A
pDex577-TbGPR89-ΔLoopTy1	This paper	N/A
pDex577-TbYJDLTy1	This paper	N/A
pDex577-TbYJDL-E388ATy1	This paper	N/A
pDex577-TbPGPTy1	This paper	N/A
pDex577-TbPOPTy1	This paper	N/A
pDex577-BIPN-PGPTy1	This paper	N/A
pDex577-BIPN-POPTy1	This paper	N/A
pET28a-TbGPR89	This paper	N/A
pET28a-YjdL	This paper	N/A
pET28a-TbGPR89-Tyr48His	This paper	N/A

**Software and Algorithms**		

GraphPad Prism	GraphPad Software	https://www.graphpad.com/scientific-software/prism/
FlowJo	Tree star software	https://www.flowjo.com/
iTASSER	Yang et al., 2015	https://zhanglab.ccmb.med.umich.edu/I-TASSER/
MUSCLE	Edgar, 2004	https://www.ebi.ac.uk/Tools/msa/muscle/
MEGA7	Kumar et al., 2016	https://www.megasoftware.net
TriTrypDB	Aslett et al., 2010	http://tritrypdb.org/tritrypdb/
GeneDB	Hertz-Fowler et al., 2004	http://www.genedb.org/Homepage
OrthoMCL	Chen et al., 2006	http://orthomcl.org/orthomcl/
Protter	Omasits et al., 2014	http://wlab.ethz.ch/protter/start/
Topcons	Tsirigos et al., 2015	http://topcons.cbr.su.se
Eukaryotic Linear Motif resource	Dinkel et al., 2016	http://elm.eu.org/infos/about.html
Pfam	Finn et al., 2016	http://pfam.xfam.org

### Contact for Reagent and Resource Sharing

Further information and requests for resources and reagents should be directed to and will be fulfilled by the Lead Contact, Keith R. Matthews (keith.matthews@ed.ac.uk).

### Experimental Model and Subject Details

#### Mice

Trypanosome infections were carried out in female healthy outbred MF1 mice at least 10 weeks old, immunocompromised with 25 mg/ml cyclophosphamide delivered intraperitoneally 24 h prior to trypanosome infection. No blinding was performed and the animals were not subject to previous procedures or drug treatment. Animal experiments were carried out according to the United Kingdom Animals (Scientific Procedures) Act under a license (PPL60/4373) issued by the United Kingdom Home Office and approved by the University of Edinburgh local ethics committee. Animals were kept in cages containing 1-5 mice on a 12h daylight cycle and maintained at room temperature. Between 2 and 3 mice were used per group; groups usually involved induction of ectopically expressed gene expression- this being activated by inclusion of doxycycline (200 μg/ml in 5% sucrose) provided in the drinking water, with control mice being provided with 5% sucrose alone. Parasitemias were estimated using a ‘rapid matching’ method involving visual comparisons of live parasites in blood by microscopy with a published standardized chart of parasite numbers per ml (Herbert and Lumsden, 1976).

#### Trypanosomes

*Trypanosoma brucei* EATRO 1125 AnTat1.1 90:13 (TETR T7POL NEO HYG) parasites were used throughout (Engstler and Boshart, 2004) for all pleomorphic cell analysis. For monomorphic trypanosome analysis, *T. brucei* Lister 427 90:13 (TETR T7POL NEO HYG) (Wirtz et al., 1999) cells were used. *In vitro*, parasites were grown in HMI-9 medium (Life technologies) (Hirumi and Hirumi, 1989) at 37°C 5% CO_2_.

### Method Details

#### Experimental design

Experiments were carried out without blinding or randomization/stratification. All figures include information on the replicate number for each experiment. Experiments were also validated in additional pilot experiments or independent replicates. Statistical methods were carried out as detailed under ‘QUANTIFICATION AND STATISTICAL ANALYSIS’ and no data were excluded. For the analysis of phenotypes 3 animals per treatment were routinely used for analysis. Our previous analyses (e.g., Mony et al., 2014) indicate that this sample size is sufficient to detect differences between cell lines and treatment groups. In the current manuscript, the visual analytical assays applied (manual scoring by microscope) to the different treatments and groups (cell cycle scoring, analysis of PAD1 staining, scoring of flagellar labeling, morphological analysis) required analyses to be limited to 2 animals per group in mixed infection experiments. P values of less than 0.05 were considered statistically significant.

#### Parasite transfection

Parasite transfection was by Amaxa nucleofection according to previous detailed methods for monomorphic (Bühlmann et al., 2015) or pleomorphic (MacGregor et al., 2013) parasites.

#### Plasmid construction and cell line generation

The *Tb*GPR89 (TriTrypDB: *Tb*927.8.1530), *Tb*PGP (TriTrypDB: *Tb*927.4.2670) and *Tb*POP (TriTrypDB: *Tb*927.10.8020) open reading frames were amplified from *T. brucei* EATRO 1125 AnTat1.1 wild-type genomic DNA with appropriate primers (Table S5) with terminal restriction sites for insertion into the pDex577-Y vector for tetracycline-inducible overexpression with an C-terminal TY epitope tag. The resulting overexpression constructs were linearized with NotI and transfected into *Trypanosoma brucei* EATRO 1125 AnTat1.1 90:13 (Pleomorphs) or Lister 427 90:13 (monomorphs) cells. Several independent cell lines were isolated and their growth analyzed *in vitro* or *in vivo* in the presence or absence of tetracycline, or doxycycline, respectively. Expression was confirmed by western blotting using an anti-TY antibody. To generate the BIP-PGP contruct, the BIP N-terminal sequence was amplified from *T. brucei* genomic DNA and subcloned into the pDEX-PGPty plasmid at the N terminus with the appropriate restriction enzymes. The Bacterial YjdL gene (*Escherichia coli* str. K-12 substr. W3110) was amplified from BL21 genomic DNA and cloned into the pDex577-Y plasmid for integration into *T. brucei* pleomorphic cells. For site directed mutagenesis, the QuikChange II Site-Directed Mutagenesis Kit was used (Agilent). For generation of conditional KOs of GPR89, *Trypanosoma brucei* EATRO 1125 AnTat1.1 90:13 were transfected with pLEW100cre-EP1 containing the Cre recombinase. The GPR89 gene was cloned into pyrFEKO-PUR plasmid (containing 5′ and 3′ GPR89 UTRs) and transfected into *T. brucei* EATRO 1125 AnTAT 90-13 Cre cells. Subsequently, the second GPR89 allele was targeted using pyrFEKO-BSD. Analysis of Cre induction was carried out according to Kim et al. (2013). Thus, induction of Cre with Doxycycline acts to remove both the BSD-TK cassette and the GPR89ty-Puro-TK allele, generating a null mutant. Null mutants were then selected by their sensitivity to blasticidin and puromycin, and resistance to gancyclovir (GCV), which counter-selects TK-expressing cells. 50 μg/ml GCV was used to select for the loss of TK.

To enable the use of CRISPR tools in *T. brucei* pleomorphic cells, we introduced into *T. brucei* EATRO 1125 Antat 1.1 cells the pJ1339 plasmid (a derivative from pJ1173, gift from Dr. Jack Sunter, Oxford Brookes University, UK; unpublished) that carries a single resistance marker, puromycin, the tet repressor, T7 RNA polymerase and Cas9 (Beneke et al., 2017). Expression of Cas9 is constitutive. To replace the endogenous copy of GPR89, the N67Q mutant was cloned into pPOTv6 using HindIII and BamHI. For gene replacement with pPOTv6-GPR89-N67Q (Blasticidin) and pPOTv7 (Hygromycin) constructs, 10^7^ cells were transfected with the PCR reactions for the two sgRNAs and two donor DNAs (combined volume approx. 100 μl) in a total volume of 250 μl.

#### Cell cycle analysis

Methanol-fixed blood smears were rehydrated in phosphate-buffered saline (PBS) for 5 min. Slides were stained with 30 μL of 4', 6-diamidino-2-phenylindole (DAPI; 10 μg/ml in PBS) for 2 min in a humidity chamber and were then washed for 5 min in PBS. Slides were then mounted with 40 μL Mowiol containing 2.5% 1, 4-diazabicyclo(2.2.2)octane (DABCO). 250-500 cells were counted per sample and per time point except where there was very low parasitaemia, where 200 cells were counted.

#### Flow cytometry

2-5x10^6^ cells were washed twice in PBS prior to fixing in 500 μL 2% formaldehyde/0.05% glutaraldehyde > 1h at 4**°**C. Cells were then washed 3x in PBS and resuspended in 2%BSA:PBS for 30 min. Cells were then resuspended in primary antibody diluted in 2%BSA:PBS (αEP procyclin (Cedar Lane laboratories) was diluted 1:500) and were incubated overnight at 4**°**C. The cells were washed twice in PBS and were resuspended in secondary antibody diluted in 2%BSA:PBS (α-mouse FITC was diluted 1:1000). The cells were washed twice in PBS and were resuspended in 500 μL PBS containing 0.02 μg/ml DAPI. Samples were then processed on an LSRII flow cytometer (BD Biosciences). Positive controls and secondary antibody only controls were included. Analysis was performed using FlowJo software (Tree Star).

#### Western blotting

An antipeptide antibody recognizing *Tb*GPR89 amino acids LDASQVSERIKSNFS was generated in rabbits (Eurogentec). For detection of GPR89, cells were resuspended in ice-cold 1 mM TLCK (*N*_α_-Tosyl-L-lysine chloromethyl ketone hydrochloride, Sigma) at 1x10^8^ cells/ml and incubated on ice for 5 minutes then incubated 37°C for a further 15 minutes, and then diluted with to 1X with 4X 8M urea loading buffer without DTT. Protein samples were resolved on SDS-PAGE gels and blotted onto nitrocellulose membrane. Primary antibody dilutions were prepared in 1% BSA/TBS and the membrane was incubated overnight. αGPR89 antibody was used at 1:1000, αBB2 antibody (Bastin et al., 1996) was used at 1:20 to detect the TY-tagged *Tb*GPR89, αPAD1 antibody (Dean et al., 2009) was used at 1:1000 and αEF1 (elongation factor 1-alpha, Merck Millipore 05-235) was used for loading controls at 1:7000. Secondary antibodies were diluted in 50% TBS and 50% Li-Cor blocking buffer. Both anti-mouse (IRDye® 680 goat anti-mouse, Li-Cor) and anti-rabbit (goat anti-rabbit IgG (H+L) Dylight 800, Thermoscientific) secondary antibodies were diluted 1:7000. Signal was detected on a Li-Cor Odyssey imaging system.

#### *In vitro* differentiation to procyclic forms

Parasites were resuspended at 2x10^6^/ml in SDM79 media (GIBCO by Life technologies) containing 6mM cis-aconitate (Sigma, A3412) and were incubated at 27**°**C. Samples were collected for flow cytometry at 0h, 3h and 6h. Progression to procyclic forms was monitored by their expression of EP procyclin using flow cytometry as detailed above.

#### MitoTracker assays

Bloodstream-form trypanosomes (2-3x10^6^/ml) were incubated in HMI-9-medium containing 100 nM MitoTracker Red CMXROS (Molecular Probes) for 30 min at 37**°**C. Then the cells were washed with HMI-9 and incubated for a further 20 min in the absence of MitoTracker, after which the parasites were fixed for 2 min at 4**°**C with 0.4% paraformaldehyde (prepared fresh in PBS). The cells were then washed once with PBS and air-dried smears were prepared. The slides were fixed for 10 min in methanol at 20**°**C, before rehydration for 10 min in PBS, followed by DAPI staining and mounting in MOWIOL.

#### Expression in *E. coli*

A single colony of *E. coli* BL21-CodonPlus (DE3)-RIPL cells containing the plasmids pET28a- (*Tb*GPR89, YjdL, *Tb*GPR89-TYR48) or empty pET28a was inoculated in 3 mL LB media containing 100 μg/mL kanamycin and 34 μg/mL chloramphenicol and allowed to grow overnight. Overnight cultures were transferred to 10 mL LB media with the same amount of antibiotics using a dilution of 1:50. The cells were allowed to grow until OD600 of 0.6–0.8 before induction with 1 mM IPTG. The cells were harvested 3 h after induction with IPTG at 37^o^C.

#### Uptake Assays with β-Ala-Lys-AMCA

Uptake assays were performed with bacteria 3 h after induction with IPTG with the fluorescent dipeptide β-Ala-Lys-AMCA (Biotrend, Cologne, Germany). Cells were harvested by centrifugation (2500 x g, 5 min) to an OD600 of 10 and incubated in Assay Buffer (33 mM HEPES, 140 mM NaCl, 5.4 mM KCl, 1.8 mM CaCl_2_, 0.8 mM MgSO_4_ and 5 mM glucose, pH 6.5) at room temperature for at least 20 min. In a final volume assay of 100 μl, 1.5 μL of a 20 mM β-Ala-Lys-AMCA stock solution (final concentration 500 μM) in the presence of absence of competing Di- or Tripeptide sublibraries, or with 40μM carbonyl cyanide m-chlorophenyl hydrazone (CCCP), was incubated with 40 μL bacteria cells at 37**°**C. Uptake was determined over 15-20 minutes. Following centrifugation and washing twice in Assay-buffer, the cell pellet was suspended in 100 μL modified Assay buffer and the uptake was quantified by fluorescence measurements (excitation at 340 nm and emission at 460 nm) on a Varioscan fluorimeter. Non-specific uptake control experiments were performed under the same conditions and procedures as described above using *E. coli* BL21-CodonPlus (DE3)-RIPL cells transformed with the empty pET28a vector.

#### Dipeptide and tripeptide sublibrary synthesis

The dipeptide and tripeptide libraries were synthesized by standard Fmoc Solid Phase Peptide Synthesis via split-and-mix. Rink Amide TentaGel beads (100 mg per sublibrary, 0.22 mmol/g, 90 μm, Rapp polymer) were used for the synthesis. The amino acids used for library production were: Ala, Arg, Asn, Asp, Gln, Glu, His, Leu, Lys, Phe, Pro, Ser, Thr, Trp, Tyr and Val. Beads were swollen in dichloromethane for 10 min prior to coupling of Fmoc deprotection. After every synthesis step, coupling or Fmoc deprotection the beads were washed with dimethylformamide and dichloromethane. A TNBS test was performed after each step. The Fmoc amino acids (3 eq) were coupled in the presence of HATU (2.9 eq) and DIPEA (6 eq) in DMF (10 ml/mg of resin) for 20 minutes and the procedure repeated twice. The Fmoc groups were removed by shaking the beads twice for 15 minutes in a solution of 20% piperidine in DMF (10 ml/g of resin). The peptides were cleaved in a solution of 95% trifuoroacetic acid (TFA), 2.5% triisopropylsilane (TIS) and 2.5% water for 4 h. The solvent was removed *in vacuo* and the samples re-dissolved in water and lyophilised. The libraries were separated in sublibraries depending on the N-terminal amino acid (2 – 11 mg) were finally dissolved in dry DMSO at 500 mM concentration. All library concentrations for growth and differentiation assays were derived from the average molecular mass of the amino acids contained.

#### Di-and Tripeptide library and peptone assays

*Trypanosoma brucei* EATRO 1125 AnTat1.1 90:13 parasites were incubated with varying concentrations of each sublibrary of di-or tripeptides (ranging from 500 μM to 62.5 μM) in 2 mL wells. The starting parasite density was 1x10^5^ parasites/ml. After 48 and 72 h, cell were counted by hematocytometer and samples were taken and fixed with 4% paraformaldehyde, and assessed for PAD1 expression. Similarly, peptone broths (Brain heart infusion, Peptone from animal proteins, Proteose peptone, Vegetable Special infusion, peptone from Gelatin - Sigma-Aldrich- or Tryptose -Fluka) were prepared according to the manufacturers’ instructions, and different concentrations tested in *T. brucei* EATRO 1125 AnTat1.1 90:13 cultures. After 48 hr, samples were taken and assessed for PAD1 expression.

#### Metabolite analysis of TbPGP or TbPOP treated human and bovine serum

Samples of human and bovine serum incubated overnight at 37°C in the presence or absence of *Tb*PGP, were centrifuged for 10 minute at 13000 g and the supernatant passed through a 3000 Da cut-off centrifugal filter membrane (Amicon Ultra-0.5). The filtrate was diluted with methanol and analyzed using an Orbitrap Mass Analyzer (80-1000 Da range, positive and negative mode). Additionally, the masses detected in each sample were screened against the monoisotopic masses of pyroglutamate and a range of commonly occurring pyroglutamate-containing serum proteins. The intensity of the free pyroglutamate signal was significantly greater in all serum samples treated with *Tb*PGPase (negative mode) while the pGlu peptides, TRH and TRH-like peptide (detected in positive mode), were present in control serum but absent from samples treated with *Tb*PGPase (highlighted cells).

For *Tb*POP, samples of human and bovine serum incubated overnight at 37°C in the presence or absence of enzyme were centrifuged for 10 minute at 13000 g and the supernatant passed through a 3000 Da cut-off centrifugal filter membrane (Amicon Ultra-0.5). The filtrate was diluted with methanol and analyzed using an Orbitrap Mass Analyzer (80-1000 Da range, positive and negative mode).

#### Structural modeling

The i-TASSER server (Yang et al., 2015) was utilized to generate a model using the sequence of *Tb*POT; chain A of structure 4IKZ was specified to be used as the template. *Tb*POT residues equivalent to template substrate-binding residues were visually identified and prioritised for mutational analysis.

#### Bioinformatic analysis

The evolutionary history of GPR89 cDNA sequences was inferred using the Neighbor-Joining method (Saitou and Nei, 1987) following multiple sequence alignment using MUSCLE (Edgar, 2004). The percentage of replicate trees in which the associated taxa clustered together in the bootstrap test (1000 replicates) are shown next to the branches in the generated trees (Felsenstein, 1981). The trees are presented to scale, with branch lengths in the same units as those of the evolutionary distances used to infer the phylogenetic tree. The evolutionary distances were computed using the Poisson correction method (Zuckerkandl and Pauling, 1965) and are in the units of the number of amino acid substitutions per site. All positions containing gaps and missing data were eliminated. Evolutionary analyses were conducted in MEGA7 (Kumar et al., 2016).

### Quantification and Statistical Analysis

Graphical and statistical analyses were carried out in GraphPad Prism version 6 (GraphPad Software, La Jolla, California, USA, https://www.graphpad.com) by two-way repeated-measures ANOVA test followed by Bonferony post hoc analysis. For individual experiments, n values are included in the Figure legend; graphs provide mean values ± SEM.

## Supplemental Information

Table S1. Metabolite Analysis of *Tb*PGP-Treated Human and Bovine Serum, Related to Figure 7 Samples of human and bovine serum treated with *Tb*PGP analyzed using an Orbitrap Mass Analyzer (80-1000 Da range, positive and negative mode). Detected masses were also screened against the monoisotopic masses of pyroglutamate and a range of commonly occurring pyroglutamate-containing serum proteins. The intensity of the free pyroglutamate signal was greater in all serum samples treated with *Tb*PGP (negative mode) while the pGlu peptides, TRH and TRH-like peptide (detected in positive mode), were present in control serum but absent from samples treated with *Tb*PGP (highlighted cells).

Table S2. Unique Metabolite Peaks Identified in *Tb*PGP-Treated Human or Bovine Serum, Related to Figure 7 Products of *Tb*PGP activity against human or bovine serum. Unique metabolite peaks identified in positive or negative ion mode, with peaks present in two or more *Tb* PGP treated samples being highlighted in red. Unambiguous identification of the product peaks is not possible without extensive further analysis.

Table S3. Metabolite Analysis of *Tb*POP-Treated Human and Bovine Serum, Related to Figure 7 Samples of human and bovine serum treated with *Tb*POP analyzed using an Orbitrap Mass Analyzer (80-1000 Da range, positive and negative mode). Sample1, *Tb*POP alone; Sample 2, Bovine serum without *Tb*POP; Sample 3, Bovine serum plus *Tb*POP; Sample 4, Human serum without *Tb*POP; Sample 5, Human serum plus *Tb*POP.

Table S4. Unique Metabolite Peaks Identified in *Tb*POP-Treated Human or Bovine Serum, Related to Figure 7 Products of *Tb*POP activity against bovine serum (POP3-Bovine serum) or human serum (POP5- Human serum). Unique metabolite peaks identified in positive or negative ion mode. Unambiguous identification of the product peaks is not possible without extensive further analysis.

Table S5. Oligonucleotides Used in This Study, Related to STAR Methods

## References

[bib1] Aslett M., Aurrecoechea C., Berriman M., Brestelli J., Brunk B.P., Carrington M., Depledge D.P., Fischer S., Gajria B., Gao X. (2010). TriTrypDB: a functional genomic resource for the Trypanosomatidae. Nucleic Acids Res.

[bib2] Bangs J.D., Brouch E.M., Ransom D.M., Roggy J.L. (1996). A soluble secretory reporter system in Trypanosoma brucei. Studies on endoplasmic reticulum targeting. J. Biol. Chem..

[bib3] Barquilla A., Saldivia M., Diaz R., Bart J.M., Vidal I., Calvo E., Hall M.N., Navarro M. (2012). Third target of rapamycin complex negatively regulates development of quiescence in Trypanosoma brucei. Proc. Natl. Acad. Sci. USA.

[bib4] Bastin P., Bagherzadeh Z., Matthews K.R., Gull K. (1996). A novel epitope tag system to study protein targeting and organelle biogenesis in Trypanosoma brucei. Mol. Biochem. Parasitol..

[bib5] Bastos I.M., Motta F.N., Charneau S., Santana J.M., Dubost L., Augustyns K., Grellier P. (2010). Prolyl oligopeptidase of Trypanosoma brucei hydrolyzes native collagen, peptide hormones and is active in the plasma of infected mice. Microbes Infect..

[bib6] Beneke T., Madden R., Makin L., Valli J., Sunter J., Gluenz E. (2017). A CRISPR Cas9 high-throughput genome editing toolkit for kinetoplastids. R. Soc. Open Sci..

[bib7] Bossard G., Cuny G., Geiger A. (2013). Secreted proteases of Trypanosoma brucei gambiense: possible targets for sleeping sickness control?. Biofactors.

[bib8] Bradford W., Buckholz A., Morton J., Price C., Jones A.M., Urano D. (2013). Eukaryotic G protein signaling evolved to require G protein-coupled receptors for activation. Sci. Signal..

[bib9] Brown S.P., Taddei F. (2007). The durability of public goods changes the dynamics and nature of social dilemmas. PLoS ONE.

[bib10] Bühlmann M., Walrad P., Rico E., Ivens A., Capewell P., Naguleswaran A., Roditi I., Matthews K.R. (2015). NMD3 regulates both mRNA and rRNA nuclear export in African trypanosomes via an XPOI-linked pathway. Nucleic Acids Res..

[bib11] Caljon G., Van Reet N., De Trez C., Vermeersch M., Pérez-Morga D., Van Den Abbeele J. (2016). The dermis as a delivery site of Trypanosoma brucei for tsetse flies. PLoS Pathog..

[bib12] Capewell P., Cren-Travaillé C., Marchesi F., Johnston P., Clucas C., Benson R.A., Gorman T.A., Calvo-Alvarez E., Crouzols A., Jouvion G. (2016). The skin is a significant but overlooked anatomical reservoir for vector-borne African trypanosomes. eLife.

[bib13] Chen F., Mackey A.J., Stoeckert C.J., Roos D.S. (2006). OrthoMCL-DB: querying a comprehensive multi-species collection of ortholog groups. Nucleic Acids Res.

[bib14] Creek D.J., Nijagal B., Kim D.H., Rojas F., Matthews K.R., Barrett M.P. (2013). Metabolomics guides rational development of a simplified cell culture medium for drug screening against Trypanosoma brucei. Antimicrob. Agents Chemother..

[bib15] Dean S., Marchetti R., Kirk K., Matthews K.R. (2009). A surface transporter family conveys the trypanosome differentiation signal. Nature.

[bib16] Deckstein J., van Appeldorn J., Tsangarides M., Yiannakou K., Müller R., Stumpf M., Sukumaran S.K., Eichinger L., Noegel A.A., Riyahi T.Y. (2015). The Dictyostelium discoideum GPHR ortholog is an endoplasmic reticulum and Golgi protein with roles during development. Eukaryot. Cell.

[bib17] Dinkel H., Van Roey K., Michael S., Kumar M., Uyar B., Altenberg B., Milchevskaya V., Schneider M., Kuhn H., Behrendt A. (2016). ELM 2016--data update and new functionality of the eukaryotic linear motif resource. Nucleic Acids Res.

[bib18] Doki S., Kato H.E., Solcan N., Iwaki M., Koyama M., Hattori M., Iwase N., Tsukazaki T., Sugita Y., Kandori H. (2013). Structural basis for dynamic mechanism of proton-coupled symport by the peptide transporter POT. Proc. Natl. Acad. Sci. USA.

[bib19] Edgar R.C. (2004). MUSCLE: multiple sequence alignment with high accuracy and high throughput. Nucleic Acids Res..

[bib20] Engstler M., Boshart M. (2004). Cold shock and regulation of surface protein trafficking convey sensitization to inducers of stage differentiation in Trypanosoma brucei. Genes Dev..

[bib21] Ernst H.A., Pham A., Hald H., Kastrup J.S., Rahman M., Mirza O. (2009). Ligand binding analyses of the putative peptide transporter YjdL from E. coli display a significant selectivity towards dipeptides. Biochem. Biophys. Res. Commun..

[bib22] Felsenstein J. (1981). Evolutionary trees from gene frequencies and quantitative characters: finding maximum likelihood estimates. Evolution.

[bib23] Finn R.D., Coggill P., Eberhardt R.Y., Eddy S.R., Mistry J., Mitchell A.L., Potter S.C., Punta M., Qureshi M., Sangrador-Vegas A. (2016). The Pfam protein families database: towards a more sustainable future. Nucleic Acids Res.

[bib24] Geiger A., Hirtz C., Bécue T., Bellard E., Centeno D., Gargani D., Rossignol M., Cuny G., Peltier J.B. (2010). Exocytosis and protein secretion in Trypanosoma. BMC Microbiol..

[bib25] Guettou F., Quistgaard E.M., Trésaugues L., Moberg P., Jegerschöld C., Zhu L., Jong A.J., Nordlund P., Löw C. (2013). Structural insights into substrate recognition in proton-dependent oligopeptide transporters. EMBO Rep..

[bib26] Gutierrez A.N., McDonald P.H. (2018). GPCRs: Emerging anti-cancer drug targets. Cell. Signal..

[bib27] Herbert W.J., Lumsden W.H. (1976). Trypanosoma brucei: a rapid “matching” method for estimating the host’s parasitemia. Exp. Parasitol..

[bib28] Hertz-Fowler C., Peacock C.S., Wood V., Aslett M., Kerhornou A., Mooney P., Tivey A., Berriman M., Hall N., Rutherford K. (2004). GeneDB: a resource for prokaryotic and eukaryotic organisms. Nucleic Acids Res.

[bib29] Hirumi H., Hirumi K. (1989). Continuous cultivation of Trypanosoma brucei blood stream forms in a medium containing a low concentration of serum protein without feeder cell layers. J. Parasitol..

[bib30] Homer C.M., Summers D.K., Goranov A.I., Clarke S.C., Wiesner D.L., Diedrich J.K., Moresco J.J., Toffaletti D., Upadhya R., Caradonna I. (2016). Intracellular action of a secreted peptide required for fungal virulence. Cell Host Microbe.

[bib31] Jackson A.P., Otto T.D., Aslett M., Armstrong S.D., Bringaud F., Schlacht A., Hartley C., Sanders M., Wastling J.M., Dacks J.B. (2016). Kinetoplastid phylogenomics reveals the evolutionary innovations associated with the origins of parasitism. Curr. Biol..

[bib32] Kelly S., Reed J., Kramer S., Ellis L., Webb H., Sunter J., Salje J., Marinsek N., Gull K., Wickstead B. (2007). Functional genomics in Trypanosoma brucei: a collection of vectors for the expression of tagged proteins from endogenous and ectopic gene loci. Mol. Biochem. Parasitol.

[bib33] Kim H.S., Li Z., Boothroyd C., Cross G.A. (2013). Strategies to construct null and conditional null Trypanosoma brucei mutants using Cre-recombinase and loxP. Mol. Biochem. Parasitol..

[bib34] Kumar S., Stecher G., Tamura K. (2016). MEGA7: Molecular Evolutionary Genetics Analysis Version 7.0 for Bigger Datasets. Mol. Biol. Evol..

[bib35] Lazazzera B.A., Grossman A.D. (1998). The ins and outs of peptide signaling. Trends Microbiol..

[bib36] MacGregor P., Szöőr B., Savill N.J., Matthews K.R. (2012). Trypanosomal immune evasion, chronicity and transmission: an elegant balancing act. Nat. Rev. Microbiol..

[bib37] MacGregor P., Rojas F., Dean S., Matthews K.R. (2013). Stable transformation of pleomorphic bloodstream form Trypanosoma brucei. Mol. Biochem. Parasitol..

[bib38] Maeda Y., Ide T., Koike M., Uchiyama Y., Kinoshita T. (2008). GPHR is a novel anion channel critical for acidification and functions of the Golgi apparatus. Nat. Cell Biol..

[bib39] Mony B.M., MacGregor P., Ivens A., Rojas F., Cowton A., Young J., Horn D., Matthews K. (2014). Genome-wide dissection of the quorum sensing signalling pathway in Trypanosoma brucei. Nature.

[bib40] Morty R.E., Bulau P., Pellé R., Wilk S., Abe K. (2006). Pyroglutamyl peptidase type I from Trypanosoma brucei: a new virulence factor from African trypanosomes that de-blocks regulatory peptides in the plasma of infected hosts. Biochem. J..

[bib41] Moss C.X., Brown E., Hamilton A., Van der Veken P., Augustyns K., Mottram J.C. (2015). An essential signal peptide peptidase identified in an RNAi screen of serine peptidases of Trypanosoma brucei. PLoS ONE.

[bib42] Newstead S. (2015). Molecular insights into proton coupled peptide transport in the PTR family of oligopeptide transporters. Biochim. Biophys. Acta.

[bib43] Omasits U., Ahrens C.H., Muller S., Wollscheid B. (2014). Protter: interactive protein feature visualization and integration with experimental proteomic data. Bioinformatics.

[bib44] Pandey S., Nelson D.C., Assmann S.M. (2009). Two novel GPCR-type G proteins are abscisic acid receptors in Arabidopsis. Cell.

[bib45] Reuner B., Vassella E., Yutzy B., Boshart M. (1997). Cell density triggers slender to stumpy differentiation of Trypanosoma brucei bloodstream forms in culture. Mol. Biochem. Parasitol..

[bib46] Roy A., Kucukural A., Zhang Y. (2010). I-TASSER: a unified platform for automated protein structure and function prediction. Nat. Protoc..

[bib47] Saitou N., Nei M. (1987). The neighbor-joining method: a new method for reconstructing phylogenetic trees. Mol. Biol. Evol..

[bib48] Saldivia M., Ceballos-Pérez G., Bart J.M., Navarro M. (2016). The AMPKα1 pathway positively regulates the developmental transition from proliferation to quiescence in Trypanosoma brucei. Cell Rep..

[bib49] Shapiro S.Z., Naessens J., Liesegang B., Moloo S.K., Magondu J. (1984). Analysis by flow cytometry of DNA synthesis during the life cycle of African trypanosomes. Acta Trop..

[bib50] Silvester E., Young J., Ivens A., Matthews K.R. (2017). Interspecies quorum sensing in co-infections can manipulate trypanosome transmission potential. Nat. Microbiol..

[bib51] Taddese B., Upton G.J., Bailey G.R., Jordan S.R., Abdulla N.Y., Reeves P.J., Reynolds C.A. (2014). Do plants contain g protein-coupled receptors?. Plant Physiol..

[bib52] Trindade S., Rijo-Ferreira F., Carvalho T., Pinto-Neves D., Guegan F., Aresta-Branco F., Bento F., Young S.A., Pinto A., Van Den Abbeele J. (2016). Trypanosoma brucei parasites occupy and functionally adapt to the adipose tissue in mice. Cell Host Microbe.

[bib53] Tsirigos K.D., Peters C., Shu N., Käll L., Elofsson A. (2015). The TOPCONS web server for consensus prediction of membrane protein topology and signal peptides. Nucleic Acids Res..

[bib54] Vassella E., Reuner B., Yutzy B., Boshart M. (1997). Differentiation of African trypanosomes is controlled by a density sensing mechanism which signals cell cycle arrest via the cAMP pathway. J. Cell Sci..

[bib55] Vincent I.M., Daly R., Courtioux B., Cattanach A.M., Biéler S., Ndung’u J.M., Bisser S., Barrett M.P. (2016). Metabolomics identifies multiple candidate biomarkers to diagnose and stage human African Trypanosomiasis. PLoS Negl. Trop. Dis..

[bib56] Wirtz E., Leal S., Ochatt C., Cross G.A.M. (1999). A tightly regulated inducible expression system for conditional gene knock-outs and dominant-negative genetics in Trypanosoma brucei. Mol. Biochem. Parasitol..

[bib57] Yang J., Yan R., Roy A., Xu D., Poisson J., Zhang Y. (2015). The I-TASSER Suite: protein structure and function prediction. Nat. Methods.

[bib58] Zimmermann H., Subota I., Batram C., Kramer S., Janzen C.J., Jones N.G., Engstler M. (2017). A quorum sensing-independent path to stumpy development in Trypanosoma brucei. PLoS Pathog..

[bib59] Zuckerkandl E., Pauling L. (1965). Molecules as documents of evolutionary history. J. Theor. Biol..

