# Supplementary material for: Oligopeptide Signaling through TbGPR89 Drives Trypanosome Quorum Sensing
Source: Cell. 2019 Jan 10;176(1-2):306–317.e16. doi: 10.1016/j.cell.2018.10.041 (PMC6333907; doi:10.1016/j.cell.2018.10.041)
Supplement: Table S5. Oligonucleotides Used in This Study, Related to STAR Methods [file mmc5.pdf]

**Oligonucleotides**		
Sequence	Amplicon	Name
TCTAGAGGATCCTCTTCTAAGGTGGGGTGTGC	pZJM-GPR89 forw	GPCR RNAi 2 F
CTCGAGCCCGGGCTAACTGCCCGACGAAGAAG	pZJM-GPR89 rev	GPCR RNAi 2 R
ACTAGTATGCTGTTGAAACTTGGCGCT	pDEX-GPR89TY forw	OL24
TCTAGATTAATCTGCGGCGGCACA	pDEX-GPR89TY rev	OL25
TCTAGAGAAGAAGGCTCCCAT	pDEX-∆415-483TY	OL32
GTCGACTATGTACGGAGTAATAA	5' UTR GPCR forw	OL59
AAGCTTAGCCGTGGGGGACGC	5' UTR GPCR rev	OL60
GGATCCTGGGGAATAACATTT	3' UTR F forw	OL61
GCGGCCGC TTGTCTCTGGCCACCCA	3' UTR rev	OL62
GGCCGGCCATGCTGTTGAAACTTGGCGCT	FORW GPCRTY for LoxP conditional (P1)	OL67
AGATCTTTAGTCAAGTGGATCTTGGTT	Rev GPCRTY for LoxP conditional (P2)	OL68
CAGCTTAGTGAGCGCATCAAGAGCCAATTTAGTATC CAGACACTGCGCTGC	GPR89 N-glycosylation site mutation forw	MUT1
GCAGCGCAGTGTCTGGATACTAAATTGGCTCTTGAT GCGCTCACTAAGCTG	GPR89 N-glycosylation site mutation rev	MUT2
AAGCTTATGAAAACACCCTCAC	pDEX-YJDL-TY forw	OL70
TCTAGAATCGTTGCTCTCCTGTATC	pDEX-YJDL-TY rev	OL71
GGATCCATGAAAACACCCTCACA	pET28a-YJDL forw	OL76
AAGCTTATCGTTGCTCTCCTGTATC	pET28a-YJDL rev	OL77
TTGTCTTCGCTTTATCGACGCACGCGTTCATCCTGC TGCT	pET28a-GPR89 TYR48 mutant forw	OL78
AGCAGCAGGATGAACGCGTGCGTCGATAAAGCGAA GACAA	pET28a-GPR89 TYR48 mutant rev	OL79
AAGCTTATGAAGCCTACAAAAC	pDEX-PGPty forw	OL80
TCTAGATTCAACTGCTTCCAT	pDEX-PGPty rev	OL81
AAGCTTATGTCGAGGATGT	BIP for pDEX-BIP+PGP forw	OL84
ACTAGTCCCGCCAACCTCGCT	BIP for pDEX-BIP+PGP rev	OL85
ACTAGTATGCGCCTCGCTTA	pDEX-POPty	OL88
TCTAGAGTCTGTCCACTGGGC	pDEX-POPty	OL89
TCTAGAACTAGTGAGGCGGGCCTGGTC	5' end gene rev for 6xHA insertion	OL55
TCTAGACGGTCGGCGGGGAGTGG	3' end gene forw for 6xHA insertion	OL56b
ACTAGTCGATCCGGACCAAGT	6xHA forw	OL57b
TCTAGAGGCAGGATCTTTACCTTG	6xHA rev	OL58
GCGCTAATGGGCTTTGCCGCCCTCTTTATTGACCCG GTG	pDEX-YjdL A388E mutant mutagenesis primer	YJDL388F
CACCGGGTCAATAAAGAGGGCGGCAAAGCCCATTA GCGC	pDEX-YjdL A388E mutant mutagenesis primer	YJDL388R
GATTACACAACAGCTAAGCGTGGCAAAGCGGTATAA TGCAGACCTGCTGCA	CRISPR cassette amplification pPOTv6	KO15300F
CCAGTAATACTTGTTGTGGCCTTCATCCCTCCAATTT GAGAGACCTGTGC	CRISPR cassette amplification pPOTv6	KO1530R:
GAAATTAATACGACTCACTATAGGTGTGAGTGAGCT GGGCTGGCGTTTTAGAGCTAGAAATAGC	Guide RNA for GPR89	1530sgRNA 5'
gaaattaatacgactcactataggTATGCTTATATGTGCTTGTG gttttagagctagaaatagc	Guide RNA for GPR89	1530sgRNA 3'
